# TRPC6 loss triggers mitochondrial dysfunction that drives white adipose tissue vulnerability to obesity and insulin resistance

**DOI:** 10.1038/s12276-026-01775-3

**Published:** 2026-07-03

**Authors:** Phan Anh Nguyen, Kyu-Hee Hwang, Duyen Thi Thuy Tran, Tae Sic Lee, Kyu-Sang Park, Seung-Kuy Cha

**Affiliations:** 1 Department of Physiology, Yonsei University Wonju College of Medicine, Wonju, Republic of Korea; 2 Department of Global Medical Science, Yonsei University Wonju College of Medicine, Wonju, Republic of Korea; 3 Organelle Medicine Research Center, Yonsei University Wonju College of Medicine, Wonju, Republic of Korea; 4 Institute of Mitochondrial Medicine, Yonsei University Wonju College of Medicine, Wonju, Republic of Korea; 5 Division of Data Mining and Computational Biology, Department of Convergence Medicine, Yonsei University Wonju College of Medicine, Wonju, Republic of Korea

**Keywords:** Fat metabolism, Mitochondria

## Abstract

Calcium signaling is essential for adipocyte function; however, the upstream Ca^2+^ channels coordinating adipose metabolism remain poorly defined. Here, we identify TRPC6 as a Ca^2+^ channel enriched in early adipocyte progenitors that orchestrate white adipose tissue (WAT) homeostasis by integrating cAMP signaling and mitochondrial bioenergetics. In *Trpc6* knockout mice, WAT emerges as the earliest and most vulnerable site of dysfunction, exhibiting pathological hypertrophic lipid accumulation despite impaired adipogenesis and intact hypothalamic signaling. Mechanistically, TRPC6 deficiency disrupts cAMP-hormone-sensitive lipase signaling and impairs mitochondrial oxidative metabolism, processes sustained by a reciprocal TRPC6–cAMP feedback loop. TRPC6 is transiently expressed during early adipogenic commitment and is indispensable for proper progenitor differentiation and lipid turnover. These findings define TRPC6 as a molecular gatekeeper that links Ca^2+^ and cAMP signaling to mitochondrial metabolism, ensuring adipocyte plasticity and energy balance. Moreover, our study reveals a previously unrecognized mechanism that underlies WAT vulnerability to metabolic dysfunction and highlights the therapeutic potential of the TRPC6–cAMP–mitochondrial axis in obesity and insulin resistance.

## TRPC6 channel links calcium and mitochondrial metabolism

This study explores the role of the TRPC6 channel in white adipose tissue metabolism, focussing on its impact on adipocyte differentiation and systemic energy balance. The authors identify TRPC6 as a key regulator of early adipocyte commitment, enriched in progenitor cells, and essential for maintaining mitochondrial function and lipolysis. Using *Trpc6* knockout mice, they demonstrate that TRPC6 deficiency leads to impaired adipocyte differentiation, mitochondrial dysfunction, and hypertrophic lipid droplet formation, resulting in systemic insulin resistance. The study uses various methods, including immunoblotting, RNA sequencing, and metabolic assays, to elucidate the molecular mechanisms involved. The findings highlight the significance of TRPC6 in coordinating Ca^2+^-cAMP signaling and mitochondrial integrity, linking it to white adipose tissue plasticity and metabolic homeostasis. This research suggests potential therapeutic targets for obesity-related metabolic disorders by modulating TRPC6 activity. "This summary was initially drafted using artificial intelligence, then revised and fact-checked by the author."

## Introduction

Obesity is a hallmark of metabolic disorders, defined by excessive adipose tissue expansion, insulin resistance, and mitochondrial dysfunction, and is a risk factor for type 2 diabetes and cardiovascular disease^1^. Although genetic and environmental factors are well recognized, Ca^2+^ signaling has emerged as a key molecular hub that integrates diverse risk factors into metabolic dysregulation^2^. Supporting this concept, increased dietary Ca^2+^ intake is associated with improved insulin sensitivity and reduced adiposity^3,4^, highlighting the importance of Ca^2+^-dependent pathways in adipose tissue regulation.

The transient receptor potential (TRP) family comprises Ca^2+^-permeable cation channels that act as cellular sensors for physical, chemical, and metabolic cues^5,6^. By transducing extracellular signals into intracellular Ca^2+^ dynamics, TRP channels orchestrate stress adaptation, energy balance, and lipid metabolism^3,5,6^. White adipose tissue (WAT) has a pivotal role in lipid storage and systemic energy homeostasis, in which tightly coordinated adipocyte differentiation and lipid turnover maintain insulin sensitivity and tissue plasticity^7,8^. Among TRP channels, TRPV4 and TRPM8 exert opposing effects on WAT: TRPV4 suppresses mitochondrial oxidation and promotes adiposity, whereas TRPM8 facilitates WAT browning and thermogenesis, thereby protecting against obesity^9,10^. Despite growing evidence linking TRP channels to adipose function^11^, the specific roles of other TRP isoforms in regulating WAT metabolism and systemic energy homeostasis remain incompletely defined.

TRPC6, a receptor-operated Ca^2+^ channel, has been extensively studied in kidney filter function, immune responses, and vascular tone regulation^12,13^. Recently, TRPC6 has emerged as a crucial regulator of metabolic health; for instance, it was shown to govern brown adipose tissue (BAT) thermogenesis via a BMPR2–p38 MAPK signaling pathway^14^. Furthermore, TRPC6 has been implicated in metabolic pathologies such as Duchenne muscular dystrophy and diabetic nephropathy, in which its deletion confers protection against lipid overload and fibrosis^15,16^. In whole-body *Trpc6* knockout (KO) mice, increased body weight and insulin resistance have been previously attributed to impaired hypothalamic energy balance and reduced pro-opiomelanocortin neuron activity^17^. Despite these insights into BAT and the central nervous system, whether TRPC6 exerts a direct, cell-autonomous function in WAT metabolism and progenitor commitment remains unclear.

Ca^2+^ signaling is known to regulate adipocyte differentiation, lipid metabolism^4,18^, and mitochondrial bioenergetics^19^. Notably, mitochondrial dysfunction and defective fatty acid oxidation (FAO) are key drivers of lipid accumulation, adipocyte hypertrophy, and insulin resistance^20^. However, the mechanistic link among TRPC6 activity, Ca^2+^ signaling, and mitochondrial function in adipocytes remains undefined.

To address this gap, we investigated the peripheral, adipocyte-intrinsic role of TRPC6 in WAT metabolism. In contrast to a prior study emphasizing hypothalamic regulatoin^17^, we identify TRPC6 as a critical regulator of early adipocyte commitment. TRPC6 is enriched in CD34-positive and Pdgfr-positive (CD34^+^/Pdfgr^+^) progenitor cells and coordinates a reciprocal Ca^2+^-cAMP signaling loop to sustain mitochondrial function and lipolysis. Loss of TRPC6 disrupts progenitor differentiation, impairs mitochondrial respiration and FAO, and promotes lipid droplet hypertrophy, ultimately leading to systemic insulin resistance. These findings uncover a previously unrecognized TRPC6-driven regulatory mechanism that links Ca^2+^ signaling to white fat plasticity and systemic metabolic homeostasis.

## Materials and methods

### Antibodies and reagents

Primary antibodies against adipogenic markers, mitochondrial proteins, and signaling molecules were used for immunoblotting, immunofluorescence, and flow cytometry. A comprehensive list of all antibodies, including manufacturers, catalog numbers, and specific working dilutions, is provided in the Supplementary information (Supplementary Table 1). All other chemical reagents were purchased from Sigma-Aldrich (St Louis, MO, USA) unless otherwise specified.

### Animals

*Trpc6* KO mice have been described previously^12^. All animal procedures were approved by the Institutional Animal Care and Use Committee of Yonsei University Wonju College of Medicine (YWC-191015-1). Details regarding housing, diet, metabolic phenotyping (CLAMS, glucose tolerance test, and insulin tolerance test), and in vivo temperature measurements are available in the Supplementary information (Supplementary Materials and Methods).

### Primary cell isolation, cell culture, and adipocyte differentiation

The primary isolated adipocytes from inguinal WAT (iWAT) and BAT were adapted from a previous publication^21^. Immortalized mouse 3T3-L1 preadipocytes and primary stromal vascular cells (SVCs) from iWAT and BAT were differentiated into mature adipocytes using a standard MDI cocktail (comprising IBMX, dexamethasone and insulin). Specific media compositions and pharmacological treatment protocols (rotenone, W7, and SS-31) are in Supplementary Materials and Methods.

### Metabolic and molecular analysis

Mitochondrial respiration (oxygen consumption rate (OCR)) and glycolysis (extracellular acidification rate (ECAR)) were measured using a Seahorse XF-96 analyzer (Agilent). Intracellular Ca^2+^ dynamics and cAMP levels were recorded using Fura-2AM and an Epac-S-H187 FRET-based biosensor, respectively. Mitochondrial ultrastructure and morphodynamics were visualized via transmission electron microscopy (HT7800, Hitachi). Comprehensive procedures for metabolic flux assays, live-cell imaging, and mitochondrial analysis are provided in Supplementary Materials and Methods.

### Adipocyte characterization and histology

Neutral lipid accumulation was assessed by Oil Red O and BODIPY staining, with quantitative biochemical analysis of triglyceride content. Tissue morphology and adipocyte size were quantified from hematoxylin and eosin-stained paraffin sections of various adipose depots. Detailed staining protocols and image analysis quantification methods are described in Supplementary Materials and Methods.

### Gene expression and transcriptome profiling

Total RNA was extracted using RiboEx, and mRNA levels were quantified by RT-quantitative PCR. For unbiased pathway analysis, bulk RNA sequencing was performed on iWAT-derived SVCs at multiple differentiation stages. Primer sequences (Supplementary Table 2) and the complete bioinformatics pipeline for RNA-seq alignment and differential expression analysis are detailed in Supplementary Materials and Methods.

### Electrophysiology and biochemical assays

TRPC6 currents were recorded in HEK293FT cells using the whole-cell patch-clamp technique. Serum leptin and intracellular cAMP concentrations were determined by enzyme-linked immunosorbent assay. Protein expression and phosphorylation states were analyzed by immunoblotting using the ChemiDoc XRS+ system. Specific buffer compositions for electrophysiology and detailed immunoassay procedures are available in Supplementary Materials and Methods.

### Statistical analysis

We analyzed the data using the GraphPad Prism software (version 9; GraphPad Software, San Diego, CA, USA). Statistical comparisons between two groups were performed using a two-tailed unpaired Student’s *t* test, whereas multiple comparisons were conducted using one-way or two-way analysis of variance, followed by a post hoc test. We considered *P* < 0.05 to indicate statistical significance. We assumed a normal data distribution, and results are presented as mean ± SEM.

## Results

### TRPC6 deficiency induces early WAT expansion and systemic insulin resistance before weight gain

**Fig. 1:**
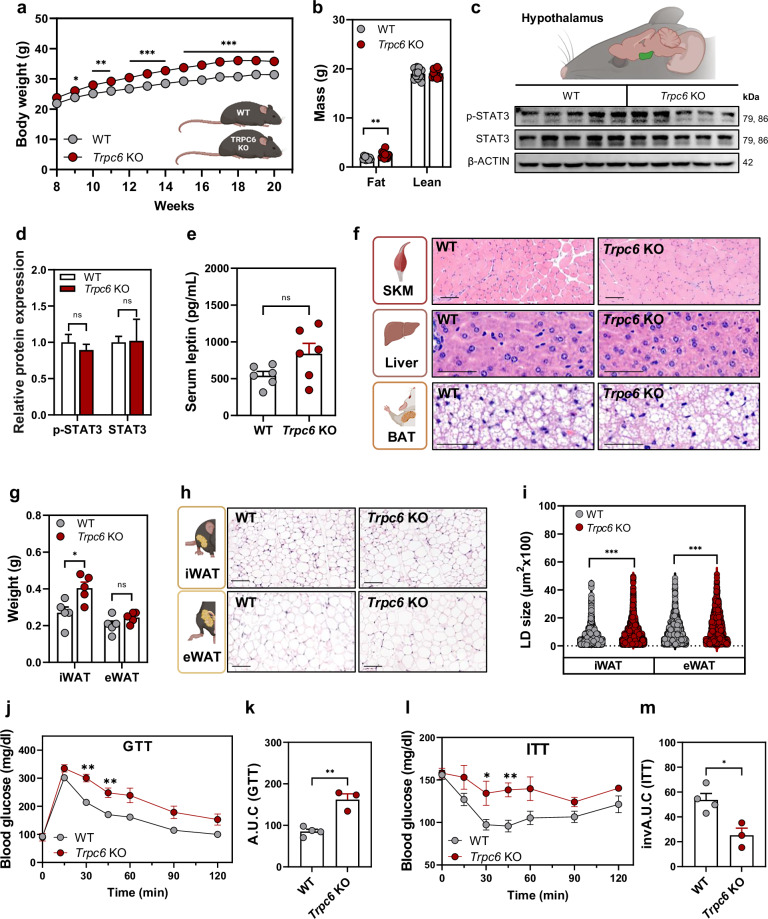
TRPC6 deficiency triggers early WAT hypertrophy and insulin resistance before weight gain. **a** Weekly body weight measurements of wild-type (WT) and *Trpc6* knockout (KO) mice from 8 weeks to 20 weeks (*n* = 19 per group). **b** NMR-based body composition analysis (*n* = 12 per group). **c**, **d** Immunoblots and quantification of p-STAT3 and STAT3 levels in the hypothalamus at 8 weeks (*n* = 5 per group). **e** Serum leptin levels in WT and *Trpc6* KO mice (*n* = 6 per group). **f** Hematoxylin and eosin staining of skeletal muscle (SKM), liver, and brown adipose tissue (BAT) at 8 weeks. Scale bar: SKM, 100 μm; liver/BAT, 50 μm. **g**, Fat depot (*n* = 10 per group). **h**, Hematoxylin and eosin-stained inguinal white adipose tissue (iWAT) and epididymal WAT (eWAT). Scale bar, 100 μm. **i** Quantification of lipid droplets (LD) in panel **h** (*n* = 5 mice per group). **j**, **k** Glucose tolerance test (GTT) at 8 weeks and quantification of the area under the curve (AUC, WT: *n* = 4, *Trpc6* KO: *n* = 3). **l**, **m** Insulin tolerance test (ITT) in 8-week-old mice and quantification of AUC (WT: *n* = 4, *Trpc6* KO: *n* = 3). Data are mean ± SEM; unpaired Student’s *t* tests for bar graphs and two-way analysis of variance for multiple time points. ns, not significant; *P < 0.05, **P < 0.01, ***P < 0.001. inv, inverse.

TRPC6 deficiency has been linked to metabolic alterations, including obesity and insulin resistance^15–17^. However, the primary initiating tissue and early molecular events remain elusive. To identify the primary site of metabolic dysregulation, we analyzed whole-body *Trpc6* KO^12^ mice (Supplementary Fig. 1a,b) at 8 weeks of age, before the onset of overt weight gain (Fig. 1a). Although body weight did not differ between *Trpc6* KO and wild-type (WT) mice at this stage, KO mice displayed a selective increase in fat mass without changes in lean mass (Fig. 1a, b), suggesting early adipose tissue expansion. Consistent with this observation, data from the Genotype-Tissue Expression Project reveal that adipose tissues exhibit among the highest levels of *TRPC6* expression across human metabolic organs (Supplementary Fig. 1c).

Given the previous emphasis on hypothalamic regulation of TRPC channels in metabolic alterations^17,22^, we first examined whether TRPC6 contributes to early fat accumulation by targeting the hypothalamus. At 8 weeks, phosphorylation of STAT3 in the hypothalamus, a downstream effector of leptin signaling^17^, was unaltered in *Trpc6* KO mice (Fig. 1c, d). Furthermore, daily food intake (Supplementary Fig. 1d) and serum leptin levels (Fig. 1e) did not differ significantly between the groups. These data indicate that central appetite regulation remains intact in young *Trpc6* KO mice and is not the primary driver of early adiposity.

Next, we evaluated the major insulin-target tissues to identify the peripheral sites affected by TRPC6 deficiency. Histological analysis revealed negligible differences in the skeletal muscle, liver, or BAT (Fig. 1f). By contrast, WAT depots were visibly enlarged in *Trpc6* KO mice (Fig. 1f), with a statistically significant increase in the weight of iWAT and a trend toward epididymal WAT (eWAT) expansion (Fig. 1g). Morphological assessment further showed that WAT adipocytes in KO mice underwent early hypertrophic remodeling characterized by enlarged lipid droplets (Fig. 1h, i), identifying WAT as the earliest and most susceptible tissue to TRPC6 loss.

Given that excessive WAT expansion drives insulin resistance^23,24^, we evaluated whether glucose homeostasis was altered in young *Trpc6* KO mice. Despite the absence of body weight differences, *Trpc6* KO mice demonstrated impaired glucose tolerance and reduced insulin sensitivity at 8 weeks of age (Fig. 1j–m), indicating that insulin resistance is tightly coupled with early WAT hypertrophy. These findings establish WAT as the primary site for early metabolic dysregulation in TRPC6-deficient mice.

### TRPC6 deficiency leads to age-dependent metabolic deterioration and progressive WAT hypertrophy

**Fig. 2:**
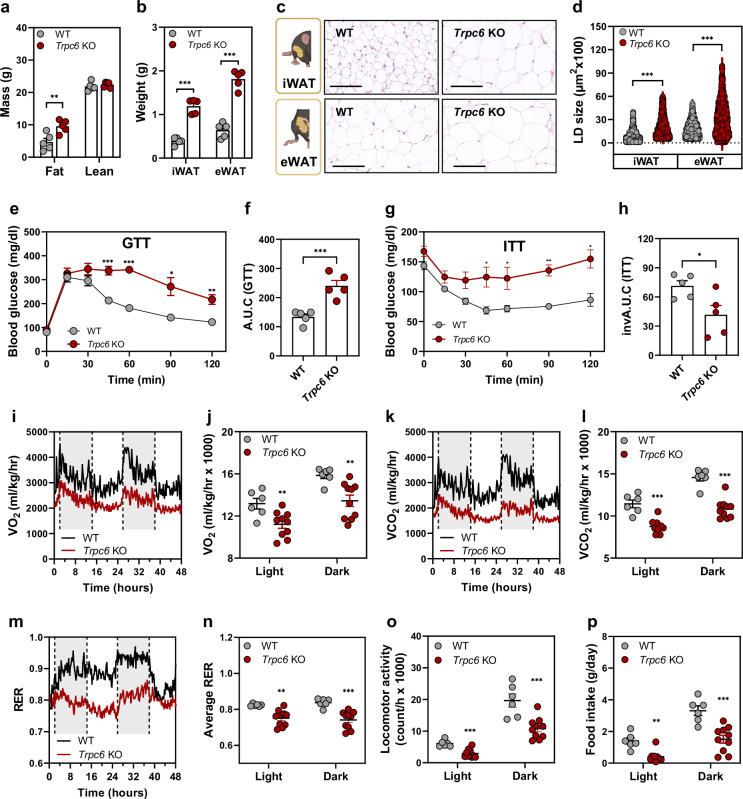
TRPC6 deficiency leads to progressive WAT hypertrophy and systemic metabolic deterioration with age. **a** NMR body composition of 20-week-old wild-type (WT) and *Trpc6* knockout (KO) mice (*n* = 5 per group). **b** Fat depot weight (*n* = 5 per group). **c** Hematoxylin and eosin staining of inguinal white adipose tissue (iWAT) and epididymal WAT (eWAT). Scale bar, 100 μm. **d** Quantification of adipocyte lipid droplet (LD) size. **e**, **f** Glucose tolerance test (GTT) and the quantitative area under the curve (AUC) (*n* = 5 per group). **g**, **h** Insulin tolerance test (ITT) and the quantitative inverse AUC (*n* = 5 per group). Metabolic cage analysis of 20-week-old mice: average oxygen consumption (VO_2_, parts **i** and **j**), carbon dioxide production (VCO_2_, parts **k** and **l**), respiratory exchange ratio (RER, parts **m** and **n**), locomotor activity (part **o**), and food intake (part **p**) across light and dark cycles (WT, *n* = 6; *Trpc6* KO, *n* = 10). Data are presented as the mean ± SEM; unpaired two-tailed Student’s *t* tests for bar graphs and two-way analysis of variance; *P < 0.05, **P < 0.01, ***P < 0.001. inv, inverse.

Expanding on previous findings that *Trpc6* deficiency induces WAT expansion and insulin resistance as early as 8 weeks of age (Fig. 1), we examined 20-week-old *Trpc6* KO mice to determine whether these early abnormalities progressively worsened. At this stage, *Trpc6* KO mice displayed significantly increased body weight and adiposity relative to WT controls, whereas lean mass remained unchanged (Fig. 2a and Supplementary Fig. 1e), confirming a selective expansion of fat mass over time.

Adipose depots expand through distinct mechanisms, depending on anatomical and physiological conditions; iWAT typically grows through hyperplasia, whereas eWAT enlarges primarily through hypertrophy^25^. In *Trpc6* KO mice, iWAT enlargement was already pronounced in the younger mice (Fig. 1g). By contrast, eWAT became the dominant expanding depot at 20 weeks (Fig. 2b), indicating an age-dependent shift toward systemic hypertrophic adipose remodeling. Histological analysis confirmed this transition, revealing adipocyte marked hypertrophy and enlarged lipid droplets in both iWAT and eWAT of *Trpc6* KO mice (Fig. 2c, d).

Given the well-established link between adiposity and insulin resistance^26^, we next assessed glucose homeostasis. *Trpc6* KO mice exhibited further aggravated glucose intolerance and reduced insulin sensitivity at 20 weeks compared with their younger counterparts (Fig. 2e–h), suggesting that TRPC6 deficiency progressively accelerates metabolic deterioration.

To evaluate the systemic energy balance, we performed indirect calorimetry. *Trpc6* KO mice demonstrated reduced oxygen consumption (VO_2_) and CO_2_ production (VCO_2_), along with a lower respiratory exchange ratio, consistent with a decreased metabolic rate and a shift toward lipid-based energy metabolism (Fig. 2i–n). Additionally, locomotor activity was diminished in KO mice (Fig. 2o), potentially contributing to the reduced energy expenditure. Despite increased adiposity, *Trpc6* KO mice consumed less food than WT control mice (Fig. 2p), likely due to significantly elevated circulating leptin levels (Supplementary Fig. 1f), which may suppress appetite via central leptin signaling. These results demonstrate that TRPC6 deficiency drives progressive WAT hypertrophy and systemic metabolic dysfunction with age, leading to overt insulin resistance and a diabetes-like phenotype.

### TRPC6 loss impairs adipocyte differentiation and promotes pathological lipid droplet hypertrophy

**Fig. 3:**
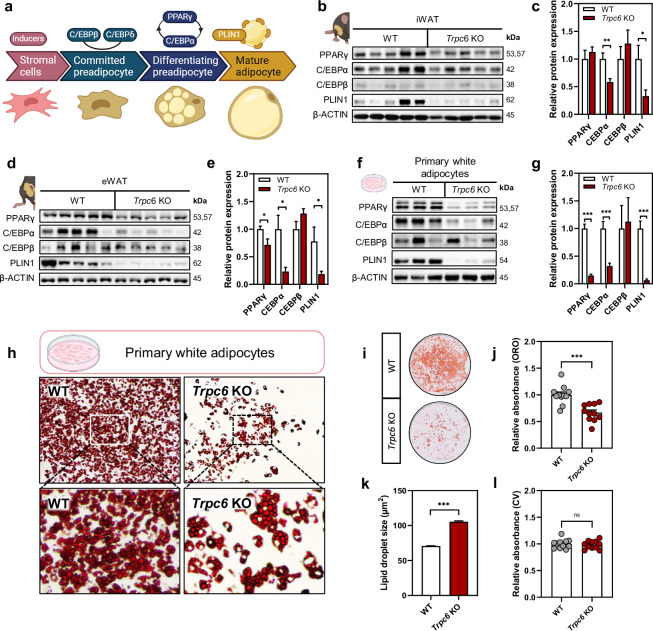
TRPC6 is required for proper adipocyte differentiation and lipid droplet remodeling in WAT. **a** Schematic illustration of adipogenesis from stromal vascular cells. Immunoblots and quantification of PPARγ, C/EBPα, C/EBPβ, and PLIN1 expression (normalized to β-actin) in inguinal white adipose tissue (iWAT) (parts **b** and **c**) and epididymal WAT (eWAT) (parts **d** and **e**) of 20-week-old mice (*n* = 5–10 per group). **f**, **g** Expression of adipogenic markers in differentiated primary white adipocytes (normalized to β-actin) (PPARγ, C/EBPα, PLIN1, *n* = 6; C/EBPβ, *n* = 3). **h**, **i** Representative images of Oil Red O (ORO)-stained wild-type (WT) or *Trpc6* KO adipocytes. Scale bar, 100 μm. **j** Quantification of relative ORO absorbance. **k** Lipid droplet size. **l** Crystal violet (CV) absorbance (*n* = 11 per group). Data represent mean ± SEM. Statistical comparisons were performed using the unpaired Student’s *t* test for bar graphs. ns, not significant; *P < 0.05, **P < 0.01, ***P < 0.001.

WAT expansion requires proper adipogenesis, a process by which SVCs (also known as progenitor cells) differentiate into mature adipocytes. This transition is marked by the induction of adipogenic markers, including C/EBPα, PPARγ, and the lipid droplet-associated protein PLIN1 (ref. ^27^) (Fig. 3a). Despite the increased iWAT and eWAT mass observed in *Trpc6* KO mice, both depots exhibited significantly reduced expression of C/EBPα and PLIN1 (Fig. 3b–e), indicating impaired adipocyte differentiation in vivo. Notably, PPARγ expression was selectively decreased in eWAT, suggesting a fat depot-specific effect of *Trpc6* deficiency on adipogenic programming.

To further investigate the regulatory role of TRPC6 in adipocyte differentiation, we isolated SVCs from subcutaneous WAT (primary white adipocytes) and induced their differentiation in vitro (Fig. 3f–l and Supplementary Fig. 2). TRPC6-deficient white adipocytes displayed markedly reduced expression of adipogenic markers (Fig. 3f, g). Oil Red O and BODIPY staining confirmed diminished overall lipid accumulation in *Trpc6* KO-derived cells (Fig. 3h–j and Supplementary Fig. 2b,c), consistent with defective differentiation.

However, a closer morphological analysis revealed a striking cellular paradox: despite the overall reduction in adipogenesis (that is, fewer mature adipocytes), those cells that did differentiate in the TRPC6-deficient group exhibited significantly enlarged, hypertrophic lipid droplets (Fig. 3k and Supplementary Fig. 2d), indicating a shift toward hypertrophic lipid storage. Importantly, cell viability remained unchanged (Fig. 3l), suggesting that TRPC6 deficiency primarily affects differentiation without inducing cytotoxicity.

Next, we validated these findings in 3T3-L1 preadipocytes using small interfering RNA targeting *Trpc6* (siTRPC6). TRPC6-depleted cells recapitulated the phenotype observed in primary adipocytes, exhibiting decreased expression of PPARγ, C/EBPα, and PLIN1, as well as reduced lipid accumulation and larger lipid droplet sizes (Supplementary Fig. 3a–g). Cell viability was unaffected (Supplementary Fig. 3h), supporting the specific role of TRPC6 in adipocyte differentiation and lipid turnover.

By contrast, TRPC6 deficiency had minimal effects on the differentiation of brown adipocytes, adipogenic marker expression, and lipid accumulation (Supplementary Fig. 4a–h), suggesting that TRPC6 primarily governs WAT-specific adipogenesis. In line with these findings, *Trpc6* mRNA expression is significantly higher in iWAT-derived and eWAT-derived SVCs than in their BAT-derived counterparts (Supplementary Fig. 4i), suggesting a more prominent functional role for TRPC6 in white versus brown adipose depots.

Adipogenesis defines both adipocyte fate and thermogenic capacity. Given the impaired adipogenesis observed in *Trpc6* KO cells, we assessed UCP1 expression as a marker of thermogenic activity. We found that UCP1 levels were significantly reduced in iWAT and primary adipocytes, whereas no significant changes were observed in BAT or eWAT (Supplementary Fig. 5a–h). The lack of effect in BAT aligns with our earlier data showing minimal TRPC6 impact in this depot, whereas the negligible change in eWAT likely reflects its inherent resistance to beiging^28^. In alignment with these findings, thermal imaging revealed a significant reduction in skin temperature, specifically in the inguinal region, but not in the suprascapular region (Supplementary Fig. 5i,j). Furthermore, *Trpc6* KO mice exhibited significantly lower core body temperatures during fasting at room temperature compared with WT controls (Supplementary Fig. 5k).

These results suggest that TRPC6 is a critical regulator of WAT adipogenesis. Loss of TRPC6 impairs adipocyte differentiation (hyperplasia) while promoting hypertrophic lipid storage, providing a mechanistic link to the pathological WAT expansion and metabolic dysfunction observed in *Trpc6* KO mice.

### TRPC6 is required for mitochondrial integrity and oxidative metabolism in white adipocytes

**Fig. 4:**
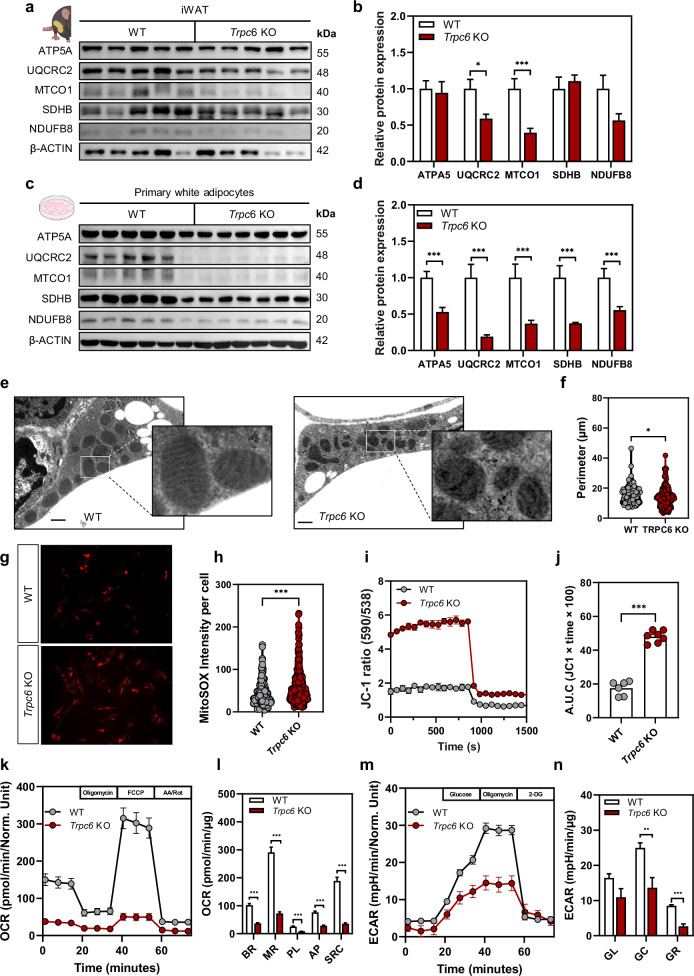
Mitochondrial dysfunction and oxidative stress accumulation in TRPC6-deficient adipocytes. Immunoblots and quantification of mitochondrial electron transport chain complex proteins in inguinal white adipose tissue (iWAT) (*n* = 5 per group) (parts **a** and **b**) and differentiated primary white adipocytes (*n* = 6 per group) (parts **c** and **d**). **e** Representative electron microscopic images of the iWAT (scale bar: 5 μm). **f** Quantification of mitochondrial perimeter, calculated from five mice per group (wild-type (WT): 9 images and *Trpc6* knockout (KO): 10 images). **g**, **h** MitoSOX-based measurement of mitochondrial reactive oxygen species production in differentiated primary white adipocytes. **i**, **j** JC-1-based assessment of mitochondrial membrane potential (WT, *n* = 6; *Trpc6* KO, *n* = 7). **k** Representative trace of the oxygen consumption rate (OCR) of WT and *Trpc6* KO differentiated primary white adipocytes (*n* = 6 per group). **l** Quantified basal respiration (BR), maximal respiration (MR), proton leak (PL), ATP-linked respiration (AP), and spare respiratory capacity (SRC) (WT, *n* = 18; *Trpc6* KO, *n* = 17). **m** Representative trace of extracellular acidification rate (ECAR) of WT and *Trpc6* KO differentiated primary white adipocytes (WT, *n* = 6; *Trpc6* KO, *n* = 3). **n** Quantification of basal glycolysis (GL), glycolytic capacity (GC), and glycolytic reserve (GR) (*n* = 5 per group). Data are mean ± SEM. Statistical comparisons were performed using an unpaired Student’s *t* test for bar graphs. *P < 0.05, **P < 0.01, ***P < 0.001. AUC, area under the curve; norm., normalized.

Mitochondria have a pivotal role in adipocyte bioenergetics, lipid oxidation, and metabolic homeostasis^29,30^. Given the impaired adipogenesis and hypertrophic lipid accumulation in TRPC6-deficient adipocytes, we investigated whether mitochondrial dysfunction contributes to these phenotypes. *Trpc6* KO mice exhibited reduced expression of electron transport chain (ETC) components (complexes I–V) in both iWAT (Fig. 4a, b) and differentiated primary white adipocytes (Fig. 4c, d), suggesting compromised mitochondrial respiration. Similar reductions were detected in eWAT (Supplementary Fig. 6a,b).

To examine the structural basis of this respiratory deficit, we performed transmission electron microscopy, which demonstrated that mitochondria in the iWAT and eWAT of *Trpc6* KO mice were fragmented and shrunken (Fig. 4e, f and Supplementary Fig. 6c,d). Consistent with these morphological changes, the expression of key mitochondrial dynamics proteins regulating fission and fusion exhibited an overall downward trend. Specifically, across iWAT and eWAT tissues, as well as primary differentiated white adipocytes, we observed a marked downregulation of both fusion (MFN1) and fission (DRP1 and MFF) mediators (Supplementary Fig. 7a–f). These results indicate a collapse of the mitochondrial network equilibrium rather than a simple shift toward one state, coupled with an overall reduction in total mitochondrial content.

Mitochondrial fragmentation has been closely linked to elevated reactive oxygen species (ROS), owing to increased electron leakage from damaged ETC complexes^31,32^. *Trpc6* KO adipocytes exhibited increased mitochondrial ROS (mtROS) levels (Fig. 4g, h). In parallel, we observed hyperpolarization of the mitochondrial membrane potential (Δ*Ψ*_m_) (Fig. 4i, j), a feature often associated with excessive ROS production and reduced electron transport efficiency^33^.

To further assess mitochondrial and glycolytic functions, we measured the OCR and ECAR. Both parameters were significantly reduced in *Trpc6* KO adipocytes (Fig. 4k–n). These deficits were recapitulated in primary white adipocytes by TRPC6 knockdown (Supplementary Fig. 8a–e), supporting a cell-autonomous role for TRPC6 in mitochondrial regulation.

These findings reveal a mechanistic role of TRPC6 in linking mitochondrial function to adipocyte fate determination. In *Trpc6* KO adipocytes, reduced mitochondrial respiration and glycolytic activity disrupted adipogenesis, whereas diminished oxidative metabolism limited lipid utilization. This imbalance favors hypertrophic lipid accumulation, contributing to the paradoxical expansion of dysfunctional adipocytes. Thus, TRPC6 functions as a critical regulator of mitochondrial fitness and adipocyte identity, thereby ensuring proper lipid turnover and maintaining WAT homeostasis under metabolic stress.

### TRPC6 is essential for mitochondrial fatty acid oxidation and adipogenic capacity in white adipocytes

**Fig. 5:**
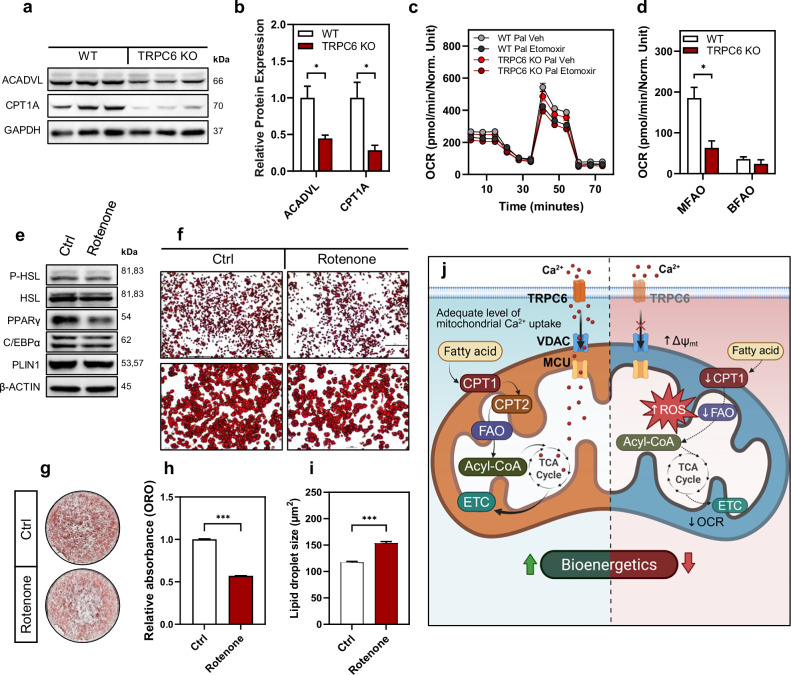
TRPC6 is essential for mitochondrial free fatty acid oxidation and lipid utilization. **a**, **b** Immunoblot and quantification of fatty acid oxidation (FAO) enzymes acyl-CoA dehydrogenase very long-chain (ACADVL) and carnitine palmitoyltransferase 1A (CPT1A) in differentiated primary adipocytes (normalized to GAPDH) (*n* = 3 per group). **c** FAO-dependent oxygen consumption rate (OCR) in response to etomoxir in differentiated primary white adipocytes (wild-type (WT): *n* = 17/10; knockout (KO): *n* = 16/10). **d** Quantification of basal and maximal FAO respiration (WT: *n* = 4; *Trpc6* KO: *n* = 5). **e** Immunoblots of lipolytic and adipogenic markers in differentiated primary white adipocytes following low-dose rotenone treatment (5 nM). **f**, **g** Representative images of Oil Red O-stained control or rotenone-treated adipocytes. Scale bar: upper panel, 500 μm; lower panel, 100 μm. **h** Quantified relative ORO absorbance of control and rotenone-treated differentiated primary white adipocytes (*n* = 6 per group). **i** Lipid droplet size in control and rotenone-treated differentiated primary white adipocytes (*n* = 3 per group). **j** Schematic model illustrating the TRPC6-mediated regulation of mitochondrial bioenergetics and FAO. Data are mean ± SEM. Unpaired Student’s *t* test; ns, not significant; *P < 0.05 and ***P < 0.001. BFAO, moderate decline in basal respiration; ETC, electron transport chain; MFAO, maximal respiration from exogenous free fatty acid; norm., normalized; ROS, reactive oxygen species; TCA, tricarboxylic acid.

FAO is a core mitochondrial process that supports energy homeostasis in adipocytes^34^. Building on the mitochondrial dysfunction observed in TRPC6-deficient adipocytes, we examined whether TRPC6 is required for FAO. Expression of essential FAO-associated enzymes, including acyl-CoA dehydrogenase very long-chain and mitochondrial fatty acid transporter (carnitine palmitoyltransferase 1A), was significantly downregulated in differentiated *Trpc6* KO primary white adipocytes (Fig. 5a, b). To assess FAO capacity directly, we measured the respiration driven by long-chain fatty acids. *Trpc6* KO adipocytes showed a marked reduction in maximal respiration from exogenous free fatty acids and a moderate decline in basal respiration, confirming impaired mitochondrial FAO (Fig. 5c, d).

To establish a causal link between mitochondrial respiratory failure and the *Trpc6* KO phenotype, we pharmacologically inhibited mitochondrial respiration using low-dose rotenone, a complex I inhibitor. Rotenone-treated WT adipocytes effectively phenocopied *Trpc6* KO cells, exhibiting reduced expression of differentiation markers and lipases (Fig. 5e). Additionally, these cells accumulated enlarged lipid droplets despite defective differentiation, providing direct evidence that mitochondrial impairment promotes pathological lipid storage and hypertrophic expansion (Fig. 5f–i). Conversely, to determine whether restoring mitochondrial integrity could mitigate the defects caused by TRPC6 loss, we utilized SS-31 — a peptide that stabilizes mitochondrial structure, scavenges ROS, and enhances ATP production^35^. Treatment with SS-31 significantly rescued the impaired adipocyte differentiation and normalized lipid droplet size observed in *Trpc6* knockdown cells (Supplementary Fig. 9a–d).

These results demonstrate that TRPC6 is a crucial regulator of mitochondrial FAO, and its loss leads to a metabolic shift in which impaired FAO facilitates excessive lipid accumulation and hypertrophic lipid droplet formation (Fig. 5j). This phenotype mechanistically links mitochondrial energy failure to the paradoxical hypertrophic lipid accumulation observed in TRPC6-deficient WAT.

### TRPC6 acts as a molecular gatekeeper, integrating Ca^2+^–cAMP signaling and lipolysis during early adipogenesis

**Fig. 6:**
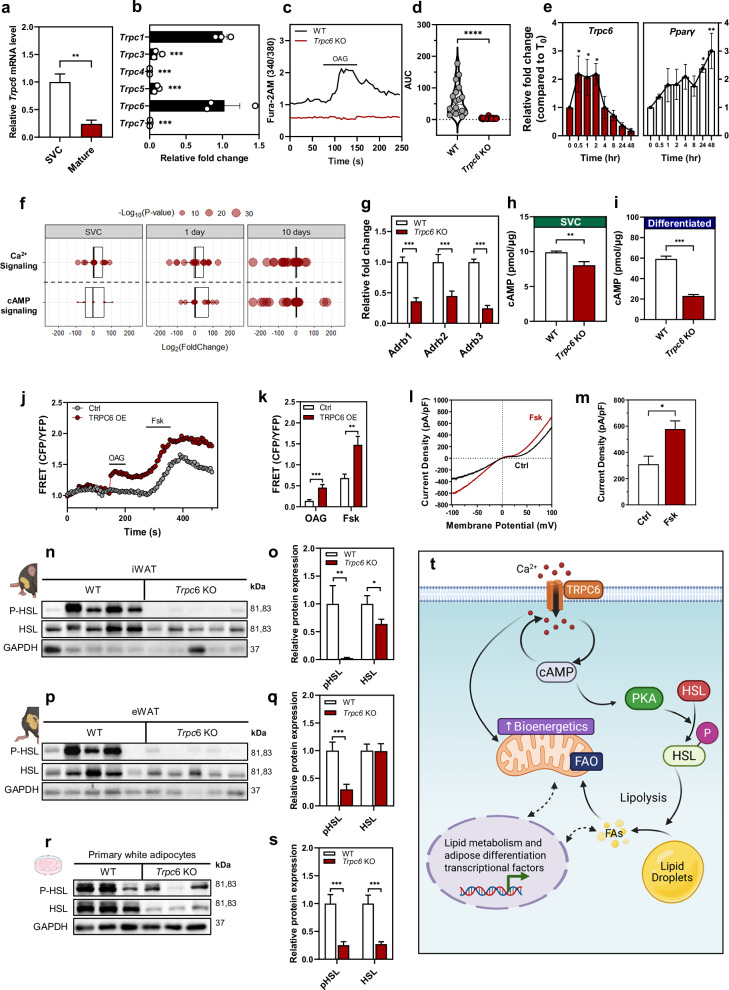
TRPC6–cAMP feedback loop coordinates adipocyte progenitor differentiation and lipid mobilization. **a** Relative mRNA levels of *Trpc6* in stromal vascular cell (SVC) and differentiated primary white adipocytes (*n* = 3 per group). **b** Heat map of relative mRNA levels of *Trpc* members in inguinal white adipose tissue (iWAT) normalized to *Trpc1* expression (*n* = 3). Traces (part **c**) and quantification (part **d**) of cytosolic Ca^2+^ measured by Fura-2AM in wild-type (WT) and *Trpc6* knockout (KO) SVCs in response to 1-oleoyl-2-acetyl- *sn*-glycerol (OAG) (*n* = 18 and 39 cells for WT and *Trpc6* KO SVCs, respectively). **e** Relative time-dependent mRNA levels of *Trpc6* and *Pparγ* in primary white adipocytes after induction (compared with *T*_0_) (*Trpc6*, *n* = 6; *Pparγ*, *n* = 5). **f** Box plot of differentially expressed genes in the cAMP signaling and Ca^2+^ signaling pathways at different time points: SVC, 1 day, and 10 days after differentiation. **g** Relative mRNA levels of *Adrb1*, *Adrb2*, and *Adrb3* in differentiated primary white adipocytes from WT and *Trpc6* KO groups (*n* = 9 per group). Cytosolic cAMP levels in SVCs (part **h**) and differentiated primary white adipocytes (part **i**). **j**, **k** FRET (Epac-S-H^187^ probe)-based assay reveals TRPC6-induced cytosolic cAMP production. Forskolin (Fsk); (*n* = 80–93 cells). **l**, **m** *I–V* curve and quantification of TRPC6 current in control and forskolin-stimulated conditions using a patch clamp (*n* = 4 per group). Representative immunoblots and quantification of p-hormone-sensitive lipase (HSL) and HSL in iWAT (parts **n** and **o**), epididymal WAT (eWAT) (parts **p** and **q**), and differentiated primary white adipocytes (parts **r** and **s**) (normalized to GAPDH) (iWAT and eWAT *n* = 10 per group and adipocytes *n* = 6 per group). **t** Schematic summary of TRPC6 function in Ca^2+^–cAMP–mitochondrial coupling during adipogenesis. Data are mean ± SEM; unpaired Student’s *t* test or two-way analysis of variance; *P < 0.05, **P < 0.01, ***P < 0.001. AUC, area under the curve; FA, fatty acid; FAO, fatty acid oxidation.

To elucidate the molecular basis of impaired adipogenesis in TRPC6-deficient adipocytes, we investigated the dynamics of TRPC6 expression and its downstream signaling pathways during adipocyte differentiation. *Trpc6* mRNA was predominantly expressed in SVCs isolated from iWAT. However, it was substantially downregulated in fully differentiated mature adipocytes (Fig. 6a). Among the TRPC family members, *Trpc6* was the second most abundant after *Trpc1* in iWAT-derived SVCs (Fig. 6b), indicating lineage-specific enrichment. To confirm TRPC6 function, we challenged isolated SVCs with the TRPC6 activators 1-oleoyl-2-acetyl- *sn*-glycerol (OAG) and endothelin-1 (ET-1). Although both ligands triggered robust cytosolic Ca^2+^ transients in WT cells, these responses were completely abolished in *Trpc6* KO counterparts (Fig. 6c, d and Supplementary Fig. 10a–c), verifying that TRPC6 is the primary mediator of Ca^2+^ entry in these progenitors.

Previous reports have identified CD34 as a marker for preadipocyte populations characterized by a high lipid-turnover rate^36^. Immunofluorescence staining confirmed that TRPC6 is co-expressed with CD34 in SVCs (Supplementary Fig. 10d,e). This association was further supported by analysis of public bulk RNA-seq data, which showed that TRPC6 is not only enriched in CD34+ SVCs but also among the upregulated genes in CD34-rich populations compared with their CD34-negative counterparts (Supplementary Fig. 10f,g). Consistent with these findings, flow cytometry showed that most TRPC6-positive cells reside within the CD34-positive fraction (Supplementary Fig. 10h). Taken together, these data suggest that TRPC6 expression is highly lineage-dependent and primarily localized to the preadipocyte population.

Time-course analysis revealed a transient increase in mRNA expression of *Trpc6* within 2 h after induction of differentiation, followed by a gradual decline (Fig. 6e, left panel). By contrast, *Pparγ* mRNA expression increased progressively (Fig. 6e, right panel), suggesting that TRPC6 functions specifically during the early commitment phase.

This stage-specific expression was further validated by human single-cell RNA-seq data from subcutaneous fat SVCs^37^, in which *TRPC6* was enriched exclusively in preadipocytes and smooth muscle cells (Supplementary Fig. 11a–e), supporting a conserved role for TRPC6 in human adipogenesis.

To identify the downstream effectors, we conducted RNA sequencing of iWAT-derived SVCs at three differentiation stages: undifferentiated baseline (day 0), early differentiation (day 1), and full differentiation (day 10) (Supplementary Fig. 11f,g). Pair-wise comparisons between WT and KO revealed 1062, 1203, and 5203 differentially expressed genes at each time point, respectively (Supplementary Fig. 11h–j). KEGG pathway enrichment revealed consistent dysregulation of cAMP and Ca^2+^ signaling pathways in *Trpc6* KO cells (Fig. 6f, Supplementary Fig. 11k–m, and Supplementary Tables 3–5). Given that β-adrenergic receptors (βARs) are key upstream regulators of cAMP production in adipocytes^38,39^, we assessed the expression of the βAR subtype in differentiated primary adipocytes. All three isoforms (*Adrb1*, *Adrb2*, and *Adrb3)* were significantly downregulated in *Trpc6* KO adipocytes (Fig. 6g), correlating with the reduced cytosolic cAMP levels observed in both SVCs and mature adipocytes (Fig. 6h, i).

Beyond adrenergic signaling, we investigated adenylyl cyclases (ADCYs), the primary regulators of cAMP. Among these, the Ca^2+^-stimulated group I isoforms (ADCY1, 3, and 8) are of particular interest^40^. We found that *Trpc6* KO-differentiated adipocytes exhibit a significant reduction in *Adcy8* expression (Supplementary Fig. 12a). To test the functional importance of this pathway, we targeted calmodulin (CaM), a key upstream regulator of ADCY8, using the inhibitor W7. Notably, W7-treated WT SVCs effectively recapitulated the *Trpc6* KO phenotype, displaying both impaired adipogenesis and the formation of enlarged, hypertrophic lipid droplets (Supplementary Fig. 12b–f), identifying the TRPC6-Ca^2+^/CaM-ADCY8 axis as a critical determinant of proper adipocyte maturation.

To determine whether TRPC6 directly modulates cAMP generation, we used a FRET-based cAMP biosensor (Epac-H-S^187^). TRPC6 activation with OAG significantly increased FRET signals, confirming TRPC6-mediated intracellular cAMP elevation (Fig. 6j, k). Furthermore, forskolin (Fsk)-induced maximal cAMP responses were amplified in TRPC6-overexpressing cells (Fig. 6k). Reciprocally, patch-clamp recordings demonstrated that Fsk augmented the TRPC6 current density (Fig. 6l, m), establishing a Ca^2+^-cAMP-positive feedback loop.

Because this signaling is essential for both adipocyte differentiation and lipase activation^39,41^, we examined hormone-sensitive lipase (HSL). Phosphorylated HSL levels were significantly reduced in both iWAT and eWAT of *Trpc6* KO mice (Fig. 6n–q), as well as in TRPC6-deficient primary adipocytes (Fig. 6r, s) and TRPC6-knockdown 3T3-L1 cells (Supplementary Fig. 13a,b). Given that HSL-mediated lipolysis provides essential fatty acid ligands for PPARγ activation^42^, its suppression likely contributes to the differentiation defects and hypertrophic lipid accumulation in *Trpc6* KO adipocytes.

Collectively, these findings indicate that TRPC6 functions as a Ca^2+^-cAMP signaling hub and molecular gatekeeper that coordinates progenitor commitment, lipolytic activity, and mitochondrial readiness during adipogenesis, thereby maintaining WAT metabolic flexibility (Fig. 6t).

## Discussion

TRPC6 is a stress-inducible Ca^2+^-permeable channel implicated in cardiovascular, renal, and metabolic disorders. Although gain-of-function mutations of TRPC6 cause glomerular and vascular defects, the physiological role of TRPC6 in adipose metabolic homeostasis remains unclear. Recently, TRPC6 was identified as an essential BAT-intrinsic regulator of metabolic health, governing brown adipose thermogenesis via a BMPR2–p38 MAPK signaling axis^14^. Although these findings underscore the critical role of TRPC6 in energy expenditure, the specific mechanisms by which it regulates WAT — the primary site for pathological expansion and insulin resistance — have not been fully elucidated. Although *Trpc6* KO mice exhibit minimal baseline abnormalities, they develop progressive obesity and insulin resistance^15,17,43,44^. This context-dependent role may be attributed to the restricted basal expression of TRPC6 and its robust induction by metabolic, oxidative, or mechanical stress^13,45^.

Our findings reveal a previously unrecognized role of TRPC6 as a peripheral regulator of WAT metabolism. In contrast to a prior report that emphasized hypothalamic regulation^17^, we identify WAT as the earliest and most vulnerable site for metabolic dysfunction in *Trpc6 KO* mice. TRPC6 is transiently enriched in CD34^+^ SVCs during the early stages of adipogenic commitment, where it orchestrates differentiation, mitochondrial metabolism, and lipid turnover to preserve adipose function and systemic insulin sensitivity.

Among the primary metabolic tissues, TRPC6 deficiency most profoundly affects WAT, supporting it as the most vulnerable tissue to TRPC6 loss. A striking paradox in our findings is that despite impaired adipogenic differentiation, *Trpc6* KO mice develop hypertrophic lipid-laden adipocytes. Typically, reduced differentiation limits lipid storage owing to the presence of fewer mature adipocytes^46^. The opposing phenotype observed in *Trpc6* KO mice suggests that the defect in adipocyte quantity (hyperplasia) is overridden by a severe decline in adipocyte quality. Specifically, the impaired mitochondrial oxidation and cAMP-mediated lipid mobilization force existing adipocytes to over-expand to store surplus energy, leading to pathological hypertrophy and systemic insulin resistance.

Intracellular Ca^2+^ ([Ca^2+^]_i_) and Ca^2+^ channels are fundamental regulators of adipogenesis, metabolism, and lipolysis^2,18,47^. Our findings identified TRPC6 as a novel plasma membrane Ca^2+^ channel required for adipogenic commitment. Unlike global [Ca^2+^]_i_ fluctuations, TRPC6-mediated Ca^2+^ influx occurs at specific developmental stages, particularly in early adipocyte progenitors (SVCs). Loss of TRPC6 impairs Ca^2+^-dependent signaling pathways crucial for early adipocyte differentiation, resulting in the downregulation of key markers, including PPARγ, C/EBPα, and PLIN1.

Mitochondria have a vital role in adipocyte energy metabolism by regulating FAO and lipogenic flux in mature adipocytes^48,49^. Our study demonstrated that *Trpc6* KO adipocytes exhibit severe mitochondrial dysfunction, with reduced expression of ETC components, fragmented mitochondria, and decreased respiratory and glycolytic capacity. Notably, although Xie et al.^14^ recently reported mitochondrial biogenesis defects in BAT, our findings demonstrate that TRPC6 is also essential for maintaining mitochondrial morphodynamics (the fission–fusion balance) in WAT. The downregulation of MFF, DRP1, and MFN1 leads to mitochondrial network collapse, resulting in a low-energy, quiescent state and elevated ROS levels. Furthermore, although TRPC6 deficiency had minimal effects on classical BAT in our model, it significantly impaired beiging capabilities in subcutaneous WAT, as evidenced by lower UCP1 expression and local temperature^28,50^.

mtROS have a pleiotropic role in adipocyte function^51^, whereas physiological ROS levels are necessary for lipogenesis and adipokine secretion. Sustained high ROS production leads to adipocyte dysfunction, hypertrophy, and insulin resistance. *Trpc6* KO adipocytes exhibited elevated mtROS levels, which paralleled the metabolic stress-induced ROS accumulation observed in the obesity model^52^. Furthermore, pharmacological inhibition of mitochondrial function with low-dose rotenone mimicked the *Trpc6* KO phenotype, supporting the notion that TRPC6 is required for mitochondrial homeostasis in adipocytes.

Ca^2+^-permeable channels have been implicated in Ca^2+^-dependent mitochondrial biogenesis and metabolic regulation^19,53^. Given that Ca^2+^ influx is a critical regulator of mitochondrial morphodynamics and bioenergetics^53,54^, our findings suggest that TRPC6 integrates Ca^2+^ signaling with mitochondrial function to maintain metabolic flexibility in WAT. Thus, TRPC6 is not only required for adipocyte differentiation but also crucial for maintaining mitochondrial integrity, ensuring proper lipid oxidation, energy balance, and insulin sensitivity. TRPC6 deficiency disrupts this balance by positioning TRPC6 at the intersection of mitochondrial dysfunction and metabolic disease, underscoring its potential as a therapeutic target for obesity-associated insulin resistance and metabolic disorders.

Intracellular cAMP signaling is a central regulator of adipocyte differentiation and lipid metabolism^41,55^ and is tightly linked with βAR-mediated and HSL-dependent lipolysis, which are crucial for lipid droplet metabolism, adipogenesis, inflammation, and insulin resistance^38,56^. Beyond its role in mitochondrial function, our analysis revealed reduced cytosolic cAMP levels and impaired βAR-HSL signaling in *Trpc6* KO adipocytes. Reduced cAMP levels impaired lipid mobilization, leading to lipid accumulation and hypertrophic growth. βAR downregulation further demonstrated the direct disruption of βAR-mediated cAMP signaling, thereby limiting the ability to mobilize stored fats efficiently. Importantly, we identified a reciprocal TRPC6–cAMP feedback loop: TRPC6-mediated Ca^2+^ influx recruits calmodulin-activated adenylyl cyclase 8 (ADCY8) to promote cAMP production, which in turn potentiates TRPC6 channel activity. Pharmacological inhibition of the upstream regulator calmodulin with W7 mimicked the *Trpc6* KO phenotype, providing a concrete molecular bridge for this axis. These findings suggest that TRPC6 is not merely a Ca^2+^ channel but also a key integrator of cAMP signaling and mitochondrial function, making it a central regulator of adipocyte function and metabolic flexibility.

Despite compelling evidence for WAT-autonomous effects, we acknowledge that using a global *Trpc6* KO model remains a limitation. However, our temporal analysis showed that WAT dysfunction precedes detectable changes in hypothalamic signaling or body weight. Future studies using adipocyte-specific models — such as adipocyte-specific *Trpc6* deletion mice or site-specific delivery of short hairpin RNA lentiviral vectors — will be essential to further delineate these roles.

In conclusion, our study identified TRPC6 as a central regulator of white adipocyte differentiation, mitochondrial fitness, and metabolic plasticity, in a tissue-specific and stage-dependent manner. Although the role of TRPC6 in BAT thermogenesis has been recently highlighted^14^, we demonstrate that it regulates a distinct Ca^2+^–cAMP signaling hub linking mitochondrial function in WAT, essential for maintaining systemic energy balance. Targeting this axis may offer new therapeutic avenues for obesity-associated metabolic disorders.

## Supplementary information

Supplementary Information

## Data Availability

The authors declare that all data supporting the findings of this study are available within the paper and its Supplementary information files. Additional data supporting the findings of this study are available from the corresponding author upon request. Public RNA-seq data analyzed in this study are available in the Gene Expression Omnibus (GEO) under accession numbers GSE129042, GSE168906, and GSE286321.
