# Supplementary material for: TRPC6 loss triggers mitochondrial dysfunction that drives white adipose tissue vulnerability to obesity and insulin resistance
Source: Exp Mol Med. 2026 Jul 3;58(7):2268–79. doi: 10.1038/s12276-026-01775-3 (PMC13434595; doi:10.1038/s12276-026-01775-3)
Supplement: Supplementary file 1 [file 12276_2026_1775_MOESM1_ESM.pdf]

Supplementary information

TRPC6 loss triggers mitochondrial dysfunction that drives white adipose tissue vulnerability to obesity and insulin resistance

Phan Anh Nguyen[1-4], Kyu-Hee Hwang[1-4]*, Duyen Thi Thuy Tran[1-4], Tae Sic Lee[3,5], Kyu-Sang Park[1-4], and Seung-Kuy Cha[1-4]*

[1]Department of Physiology, Yonsei University Wonju College of Medicine, Wonju, Republic of Korea. [2]Department of Global Medical Science, Yonsei University Wonju College of Medicine, Wonju, Republic of Korea. [3]Organelle Medicine Research Center, Yonsei University Wonju College of Medicine, Wonju, Republic of Korea. [4]Institute of Mitochondrial Medicine, Yonsei University Wonju College of Medicine, Wonju, Republic of Korea. [5]Division of Data Mining and Computational Biology, Department of Convergence Medicine, Yonsei University Wonju College of Medicine, Wonju, Republic of Korea.

TABLE OF CONTENTS

Supplementary Fig. 1 Trpc6 KO mice metabolic phenotype 3

Supplementary Fig. 2 Trpc6 Knockout differentiated adipocytes showed reduced adipogenesis and hypertrophy lipid droplet formation.. 4

Supplementary Fig. 3 Trpc6 knockdown impairs adipogenic differentiation in 3T3-L1 cells. 5

Supplementary Fig. 4 TRPC6 deficiency has minimal effect on brown adipocyte differentiation 6

Supplementary Fig. 5 TRPC6 Deficiency reduced beiging in white adipose tissues. 8

Supplementary Fig. 6 Mitochondrial ETC protein expressions and morphology in eWAT of Trpc6 KO mice 10

Supplementary Fig. 7 Mitochondrial ETC Protein Expressions and Morphology in eWAT of Trpc6 KO Mice. 11

Supplementary Fig. 8 Mitochondrial respiration and glycolytic flux are impaired in TRPC6-deficient adipocytes 12

Supplementary Fig. 9 SS-31 partially rescued Trpc6 Knockdown-dependent malfunction adipogenesis in primary white adipocytes. 13

Supplementary Fig. 10 TRPC6 is co-expressed with CD34 in preadipocytes linage from SVCs. 14

Supplementary Fig. 11 Transcriptomic profiling reveals Ca2+ and cAMP pathway dysregulation in Trpc6 KO progenitors 16

Supplementary Fig. 12 Adcy8 is altered in Trpc6 KO adipocyte and its upstream inhibitor-W7 reduces adipogenesis and causes hypertrophic lipid droplet formation. 17

Supplementary Fig. 13 HSL activation is impaired in TRPC6-depleted 3T3-L1 adipocytes 18

Supplementary Table 1. List of antibodies 19

Supplementary Table 2. Primer list 20

Supplementary Table 3. Top 20 significantly enriched KEGG pathways in the SVC group Trpc6 KO vs WT 21

Supplementary Table 4. Top 20 significantly enriched KEGG pathways in 1 day differentiated group Trpc6 KO vs WT 22

Supplementary Table 5. Top 20 significantly enriched KEGG pathways in 10 days differentiated group Trpc6 KO vs WT 23

SUPPLEMENTARY MATERIALS AND METHODS 24

REFERENCES 37

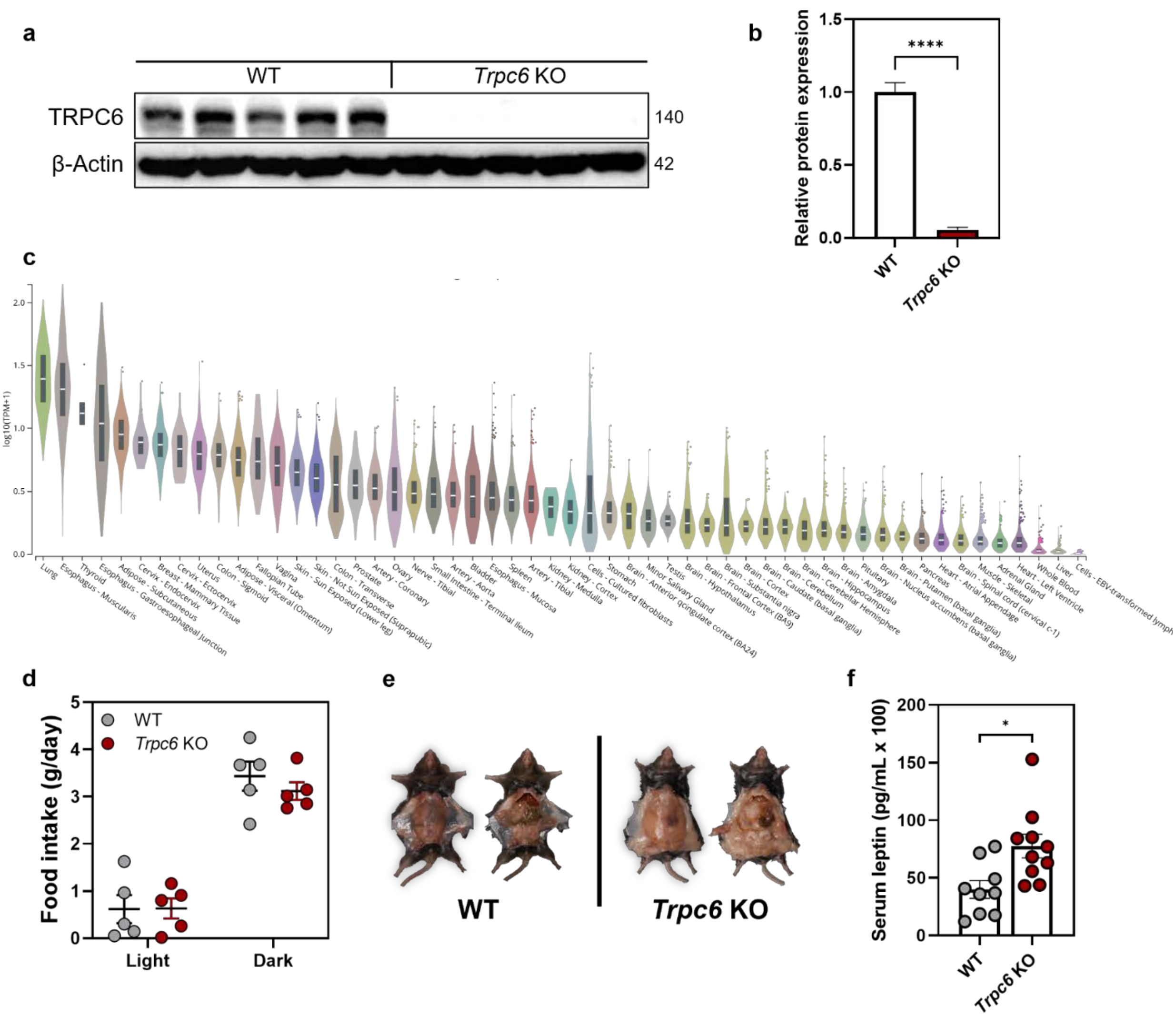

Supplementary Fig. 1 Trpc6 KO mice metabolic phenotype. a, b Immunoblot and relative quantification of TRPC6 expression in lung tissue from WT and *Trpc6* KO mice (*n* = 5 per group) **c** Bulk tissue TRPC6 expression from Gene-Tissue Expression (GTEx) Project. **d** Average food intake per every light and dark phase of 8-week-old WT and *Trpc6* KO mice (*n* = 5 per group). **e** Representative 20-week-old mice for WT and *Trpc6* KO group depicting gross images of abdominal fat. **f** Serum leptin levels of 20-week-old WT and *Trpc6* KO mice (WT: *n* = 9, TRPC6 KO *n* = 10), Data are mean ± SEM. Statistical comparisons are done using Student's *t*-test for bar graphs. *$p < 0.05$.

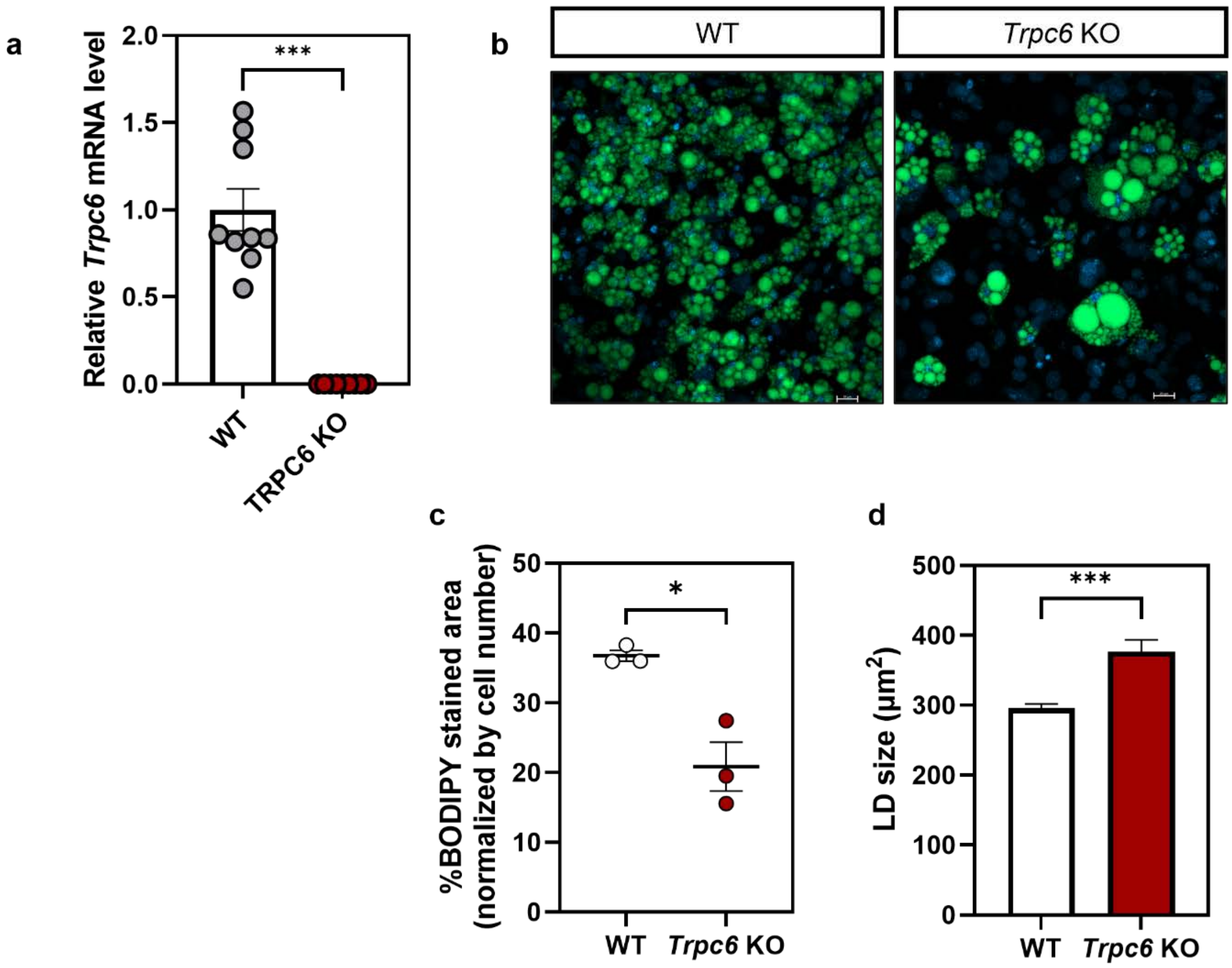

Supplementary Fig. 2 Trpc6 Knockout differentiated adipocytes showed reduced adipogenesis and hypertrophy lipid droplet formation. a Relative mRNA expression of Trpc6 in WT and Trpc6 KO primary white (n = 9 per group). **b** Representative BODIPY staining of differentiated primary white adipocytes. Scale bar: 20 μm. **c** Percentage of stained BODIPY area normalized by cell number (DAPI) (n = 3). **d** Quantification of lipid droplet size (n = 6,533 for WT and 2,181 droplets for Trpc6 KO). Data are mean ± SEM. Statistical comparisons are done using Student's *t*-test for bar graphs. *$p < 0.05$, ***$p < 0.001$.

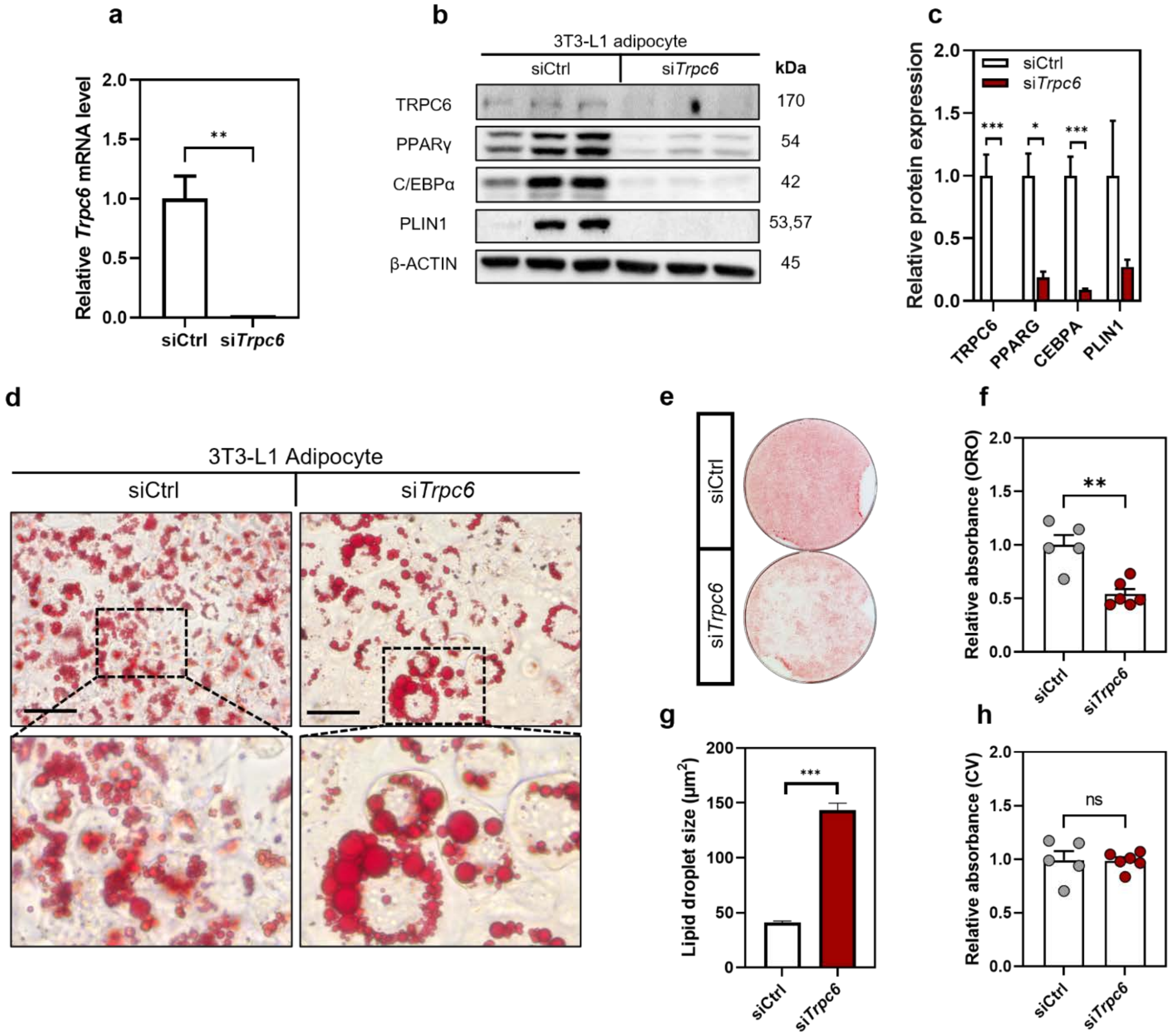

Supplementary Fig. 3 Trpc6 knockdown impairs adipogenic differentiation in 3T3-L1 cells. a Relative mRNA expression of *Trpc6* in control (siCtrl) and *Trpc6* knockdown (si*Trpc6*) 3T3-L1 cells (n = 3 per group). **$P < 0.01$. **b**, **c** Immunoblot and relative quantification of TRPC6, PPARγ, C/EBPα, and PLIN1 in differentiated control and *Trpc6* knockdown 3T3-L1 cells (n = 3 per group). **d-h** Representative oil red o (ORO) – stained images (**d**, **e**), quantified relative ORO absorbance (**f**), lipid droplet size quantification (**g**), and quantified relative crystal violet (**h**) of control and *Trpc6* knockdown differentiated 3T3-L1 cells. Scale bar: 50 μm. (siCtrl: n = 5, si*Trpc6*: n = 6). Data are mean ± SEM. Statistical comparisons are done using Student's t-test for bar graphs. *$p < 0.05$, **$p < 0.01$, ***$p < 0.001$.

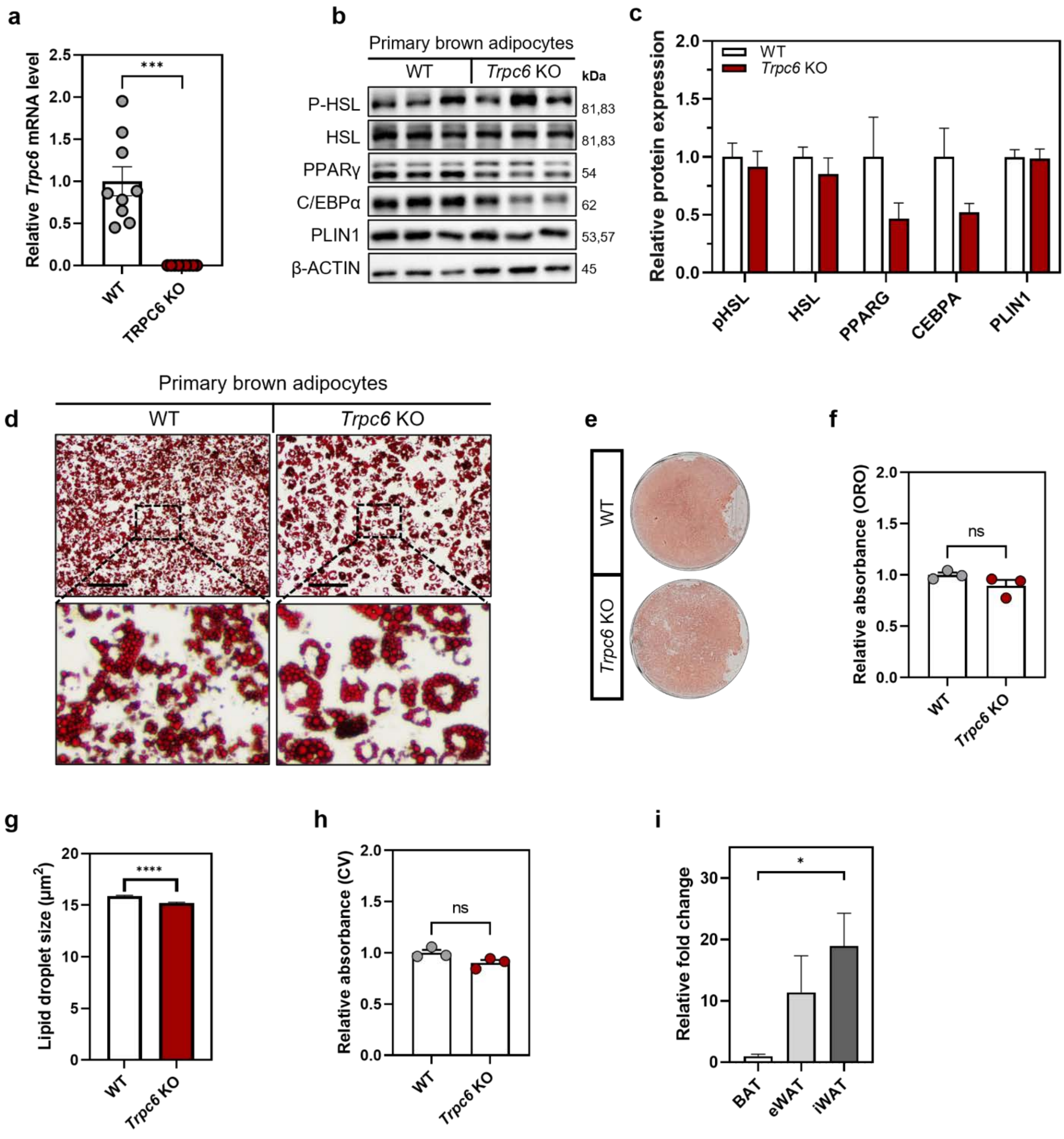

Supplementary Fig. 4 TRPC6 deficiency has minimal effect on brown adipocyte differentiation. a Relative mRNA expression of *Trpc6* in WT and *Trpc6* KO differentiated primary brown adipocytes (*n* = 9 per group). **b**, **c** Immunoblot and relative quantification of P-HSL, HSL, PPARγ, C/EBPα, and PLIN1 in WT and *Trpc6* KO differentiated primary brown adipocytes (*n* = 5 per group). **d-h** Representative Oil Red O (ORO) – stained images (**d**, **e**), quantified relative ORO absorbance (**f**), lipid droplet size quantification (**g**), and quantified relative crystal violet (**h**) of differentiated WT and *Trpc6* KO primary brown adipocytes (*n* = 3 per group). Scale bar: 200 μm (*n* = 3 per group). **i** Relative mRNA expression of *Trpc6* in SVC

from different fat depots (BAT, eWAT, and iWAT) (n = 3 per group). Data are mean ± SEM. Statistical comparisons are done using Student's *t*-test for bar graphs. ns represent not significant, $^{**}P < 0.01$, $^{***}P < 0.001$.

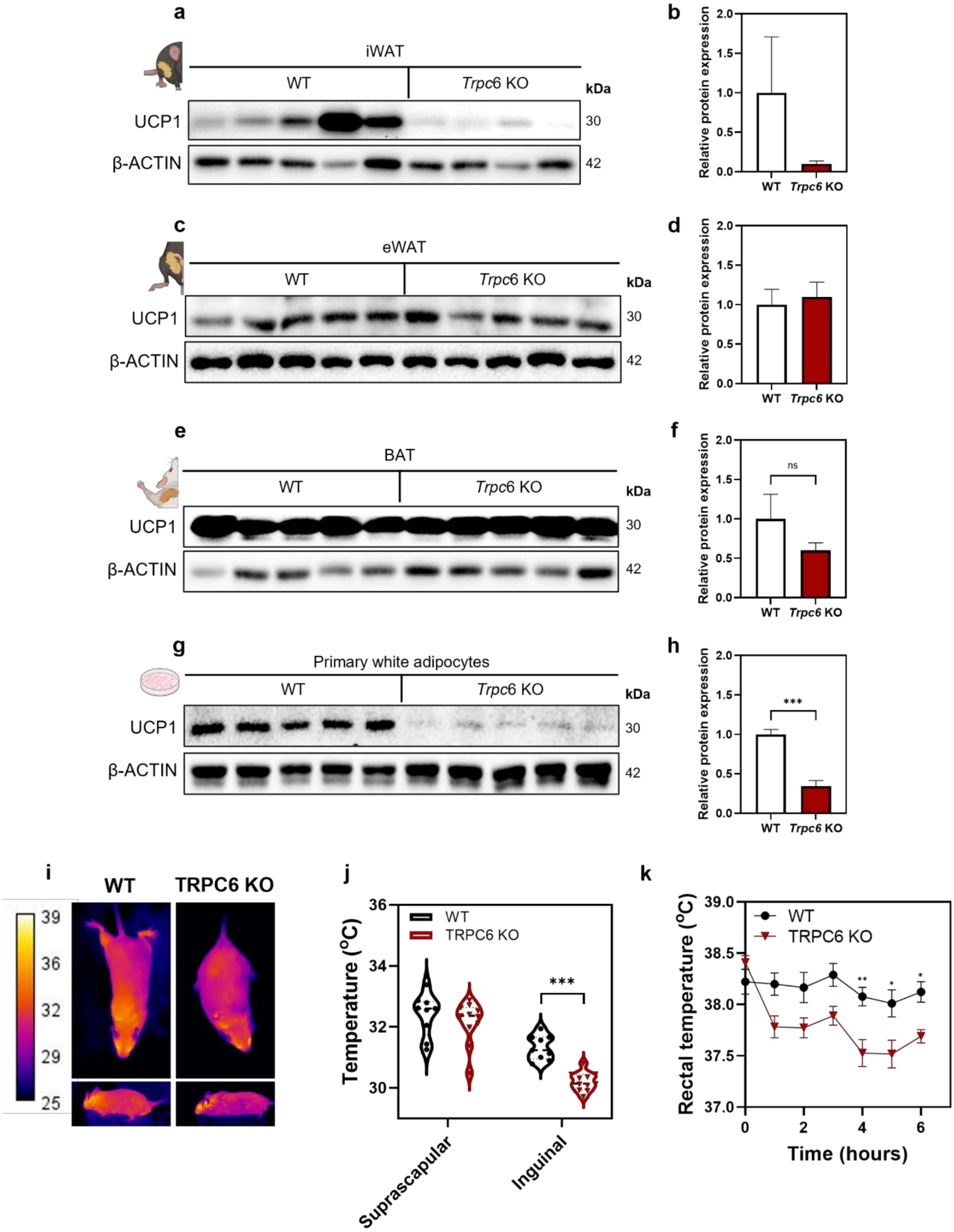

Supplementary Fig. 5 TRPC6 Deficiency reduced beigeing in white adipose tissues.

a-h Immunoblot and relative quantification of UCP1 expression in 20-week-old WT and *Trpc6* KO iWAT (**a**, **b**) (*n* = 5 and 4 for WT and *Trpc6* KO, respectively), eWAT (**c**, **d**) (*n* = 5 per

group), BAT (**e**, **f**) (*n* = 5 per group), and differentiated primary white adipocytes (**g**, **h**) (*n* = 5 per group). **i, j** Representative and quantification of skin temperature images from 20-week-old WT and *Trpc6* KO mice (*n* = 9 and 11 for WT and *Trpc6* KO, respectively). **k** Rectal temperature of 20-week-old WT and *Trpc6* KO mice (*n* = 9 and 11 for WT and *Trpc6* KO, respectively). Data are mean ± SEM. Statistic comparisons are done using Student's *t-test* for bar graphs. *$P < 0.05$, **$P < 0.01$, ***$P < 0.001$.

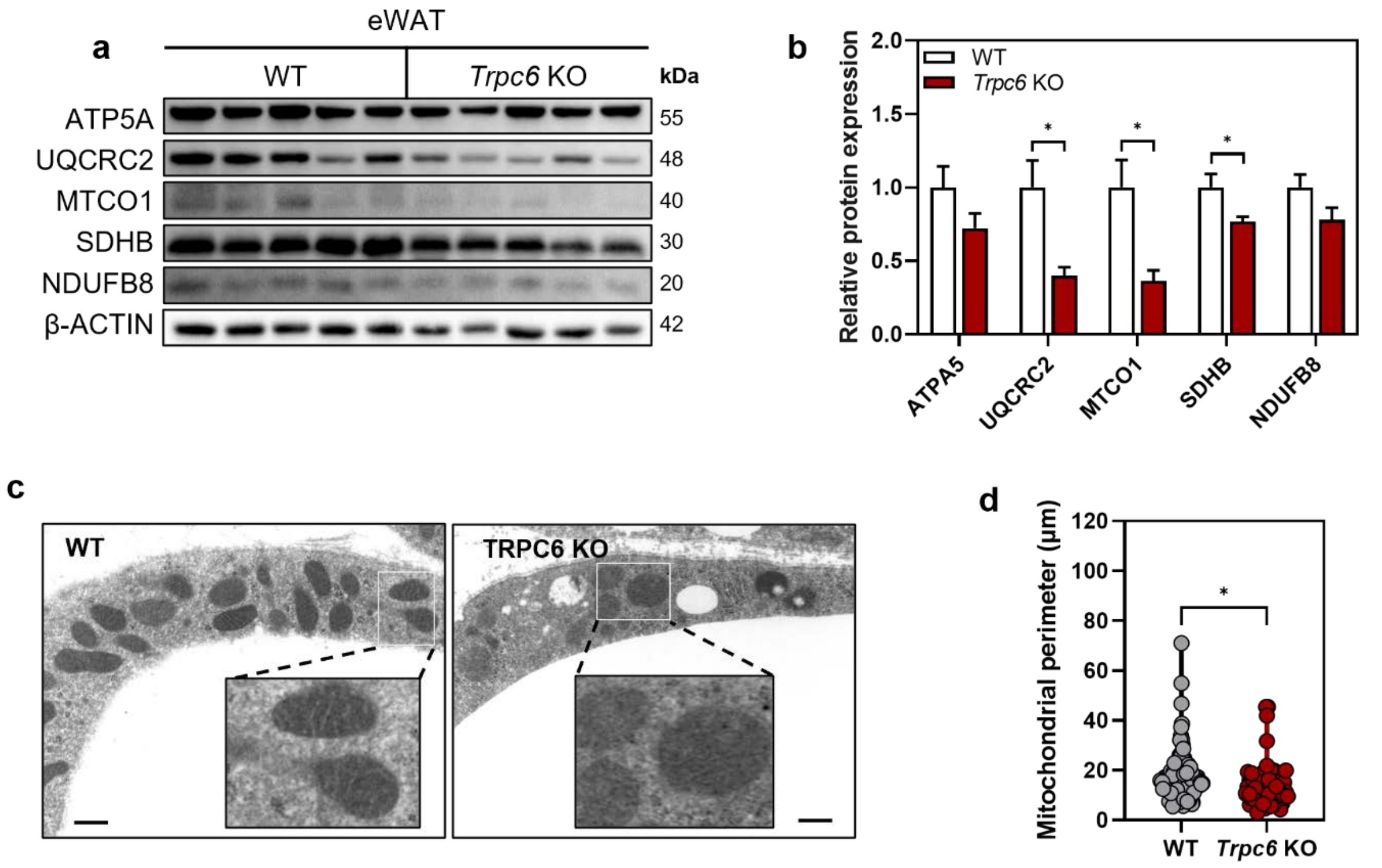

Supplementary Fig. 6 Mitochondrial ETC protein expressions and morphology in eWAT of Trpc6 KO mice. a, **b** Immunoblot and relative quantification of mitochondrial ETC complexes proteins (ATP5A, UQCRC2, MTCO1, SDHB, NDUFB8) in 20-week-old WT and *Trpc6* KO eWAT (*n* = 5 per group). **c** Representative electron microscope images of 8-week-old WT and *Trpc6* KO eWAT tissues. Scale bar: 5 μm. **d** Quantification of mitochondrial perimeter, calculated from five mice per group (WT: 10 images, *Trpc6* KO: 9 images). Data are mean ± SEM. Statistical comparisons are done using Student's *t*-test for bar graphs. *$p < 0.05$.

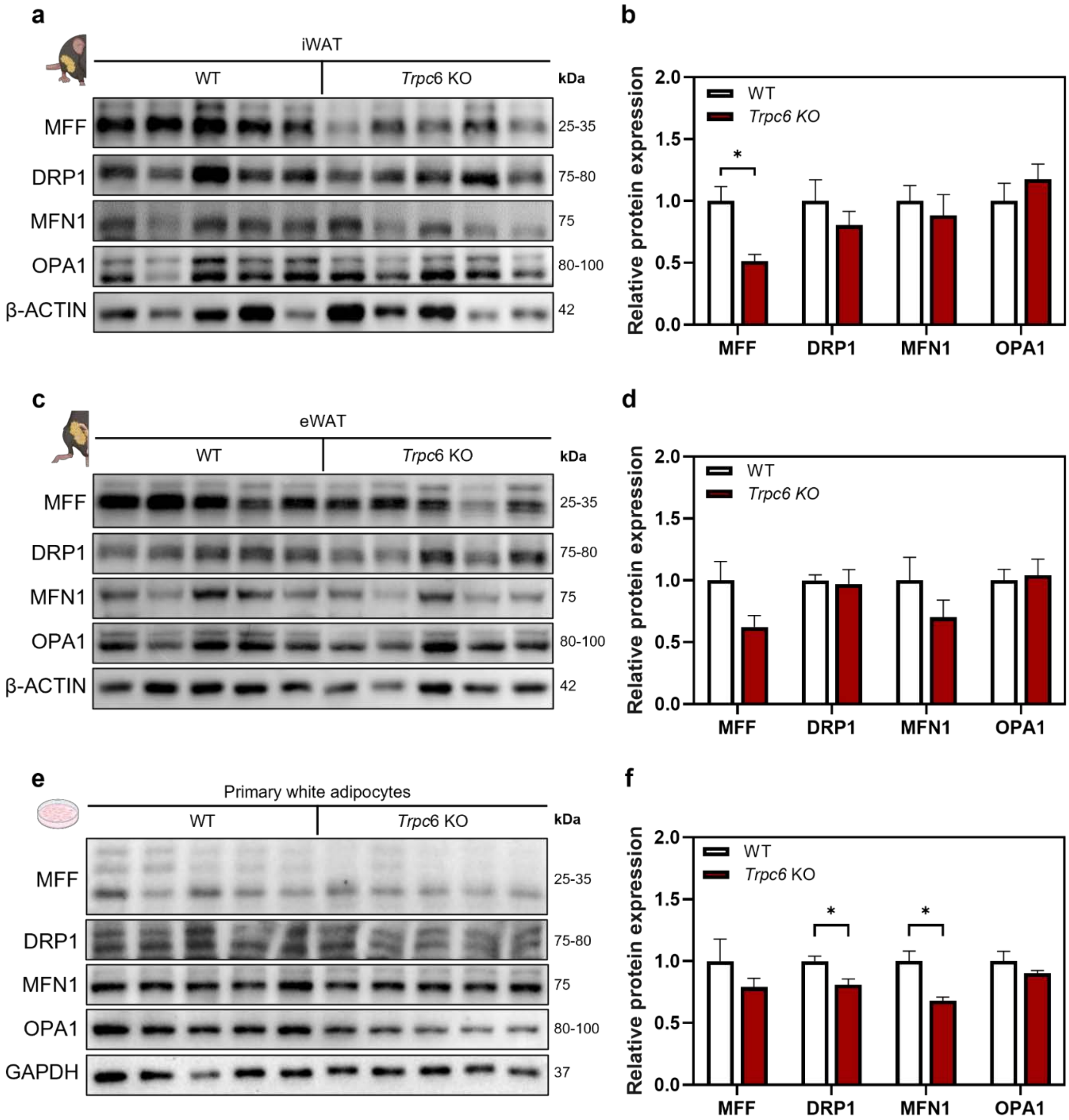

Supplementary Fig. 7 Mitochondrial ETC Protein Expressions and Morphology in eWAT of Trpc6 KO Mice. a, **b** Immunoblot and relative quantification of mitochondrial dynamics complexes proteins (MFF, DRP1, MFN1, OPA1) in 20-week-old WT and *Trpc6* KO iWAT (**a**, **b**), eWAT (**c**, **d**), and differentiated primary white adipocytes (**e**, **f**) (n = 5 per group). **d** Quantification of mitochondrial perimeter, calculated from from five mice per group (WT: 10 images, *Trpc6* KO: 9 images). Data are mean ± SEM. Statistic comparisons are done using Student's *t-test* for bar graphs. *$p < 0.05$.

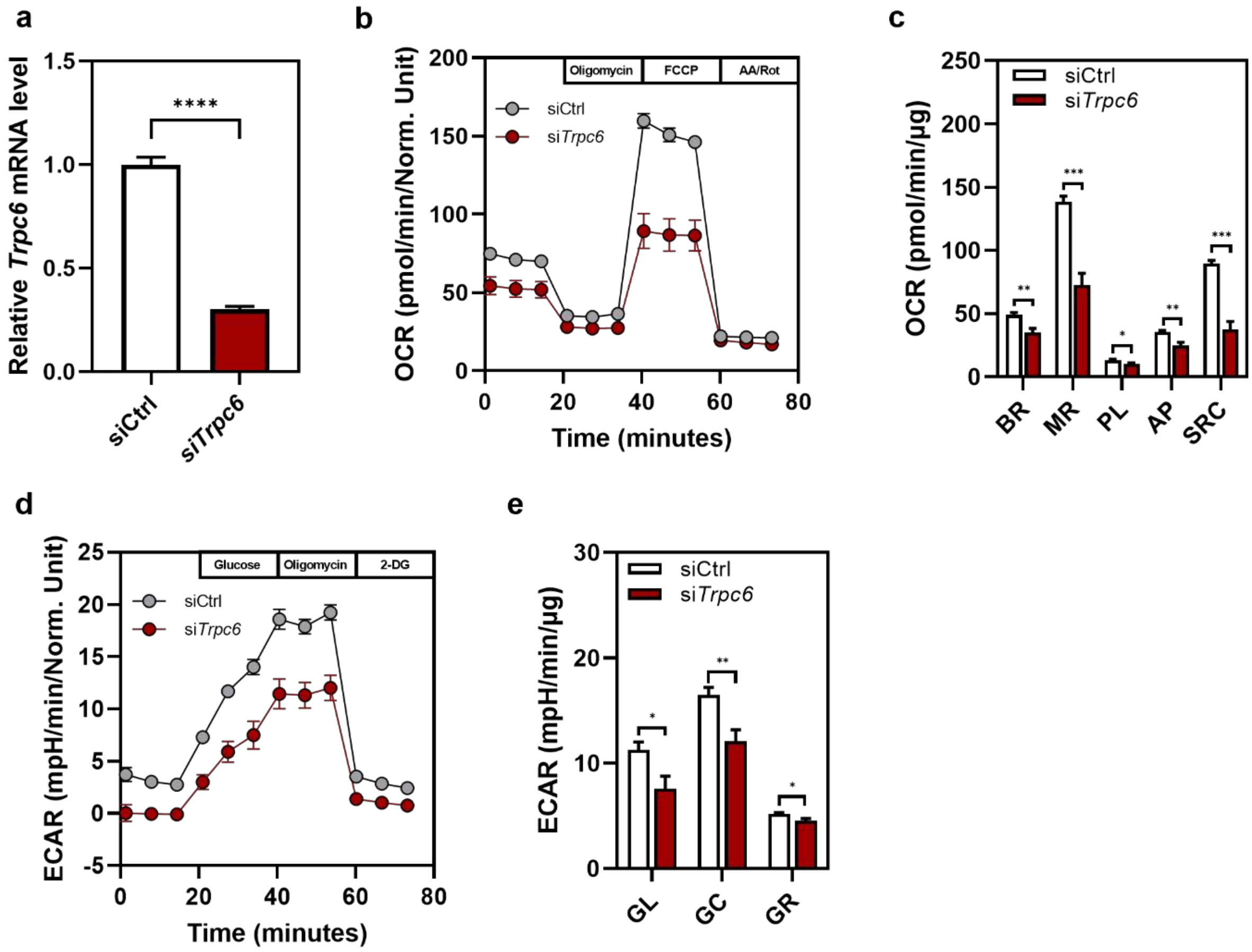

Supplementary Fig. 8 Mitochondrial respiration and glycolytic flux are impaired in TRPC6-deficient adipocytes. a Relative *Trpc6* mRNA expression in control (siCtrl) and *Trpc6* knockdown (si*Trpc6*) differentiated primary white adipocytes (*n* = 3 per group). **b** Oxygen consumption rate of control and *Trpc6* knockdown primary white adipocytes (*n* = 5 per group). **c** Quantified basal respiration (BR), maximal respiration (MR), proton leak (PL), ATP-linked respiration (AP), and spare respiration capacity (SRC) (*n* = 5 per group). **d** Extracellular acidification rate of control and *Trpc6* knockdown primary white adipocytes. *n* = 5. **e** Quantified basal glycolysis (GL), glycolytic capacity (GC), and glycolytic reserve (GR) (*n* = 5 per group). Data are mean ± SEM. Statistical comparisons are done using Student's *t*-test for bar graphs. **P* < 0.05, ***P* < 0.01, ****P* < 0.001.

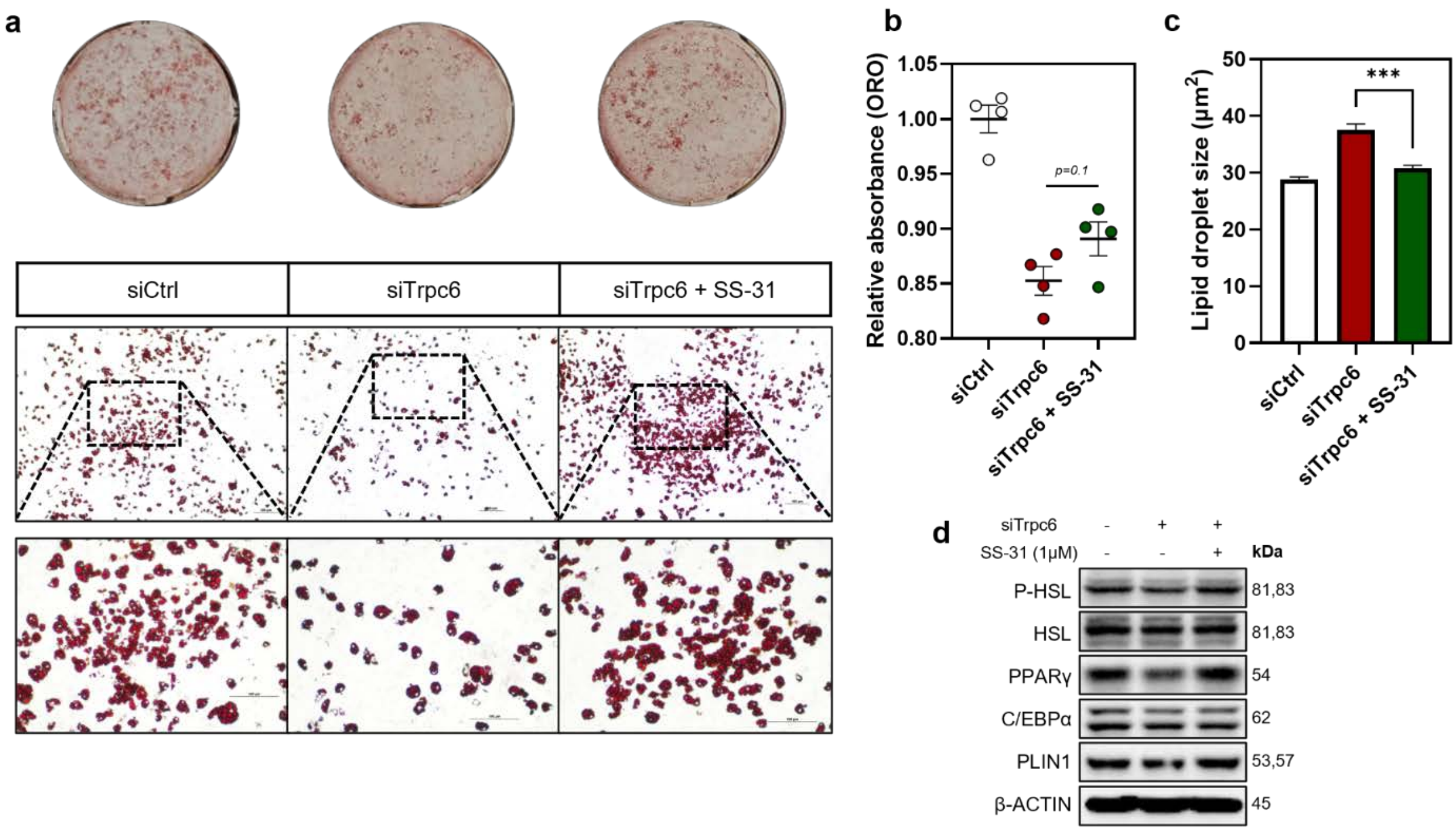

Supplementary Fig. 9 SS-31 partially rescued Trpc6 knockdown-dependent maladaptive adipogenesis in primary white adipocytes. a-c Representative Oil Red O (ORO) – stained images (**a**), quantified relative ORO absorbance (**b**), lipid droplet size quantification (**c**) of control and *Trpc6* knockdown differentiated primary white adipocytes (n = 4 per group). Scale bar: 100 μm. **d** Immunoblot of P-HSL, HSL, PPARγ, C/EBPα, and PLIN1 in siControl, *Trpc6* knockdown, and *Trpc6* knockdown with SS-31 co-treatment in differentiated primary white adipocytes. Data are mean ± SEM. Statistical comparisons are done using Student's t-test for bar graphs. ***$p < 0.001$.

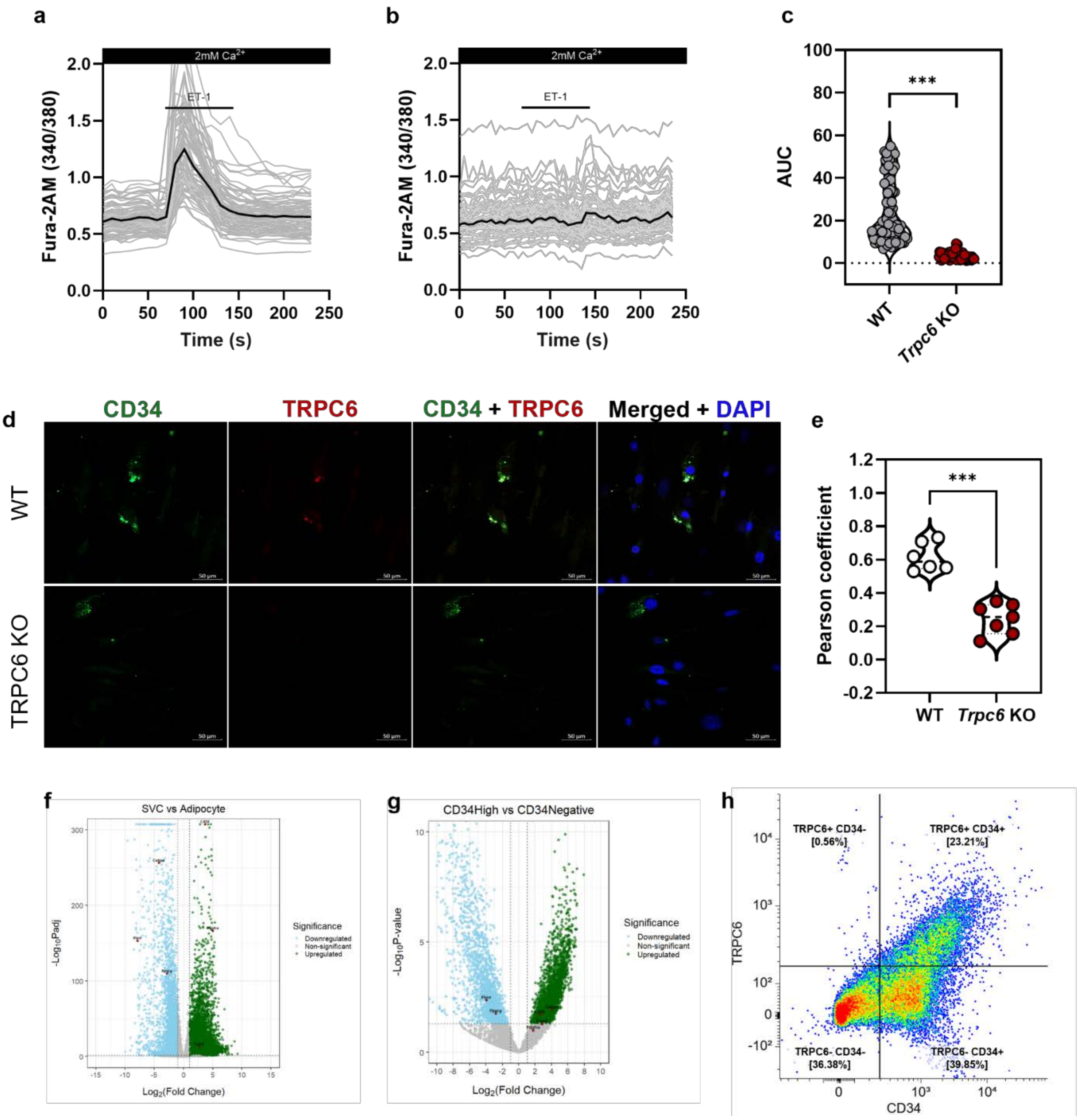

Supplementary Fig. 10 TRPC6 is co-expressed with CD34 in preadipocyte lineage from SVCs. a-c Traces (**a**, **b**) and quantification (**c**) of cytosolic Ca^{2+} measured by Fura-2AM in WT and *Trpc6* KO SVCs in response to Endothelin-1 (*n* = 60 and 82 cells for WT and *Trpc6* KO SVCs, respectively). **d** Representative immunostaining of TRPC6 (red) and CD34 (green) in WT and *Trpc6* KO SVCs. Scale bar: 50µm. **e** Pearson's correlation of TRPC6 and CD34 fluorescent signal (*n* = 6 and 7 for WT and *Trpc6* KO SVCs, respectively). **f, g** Volcano plot showing differentially expressed genes by bulk RNA-seq analysis between murine SVCs (**f**) vs Adipocyte and between CD34-high and CD34-negative populations of preadipocyte lineage (**g**). **h** Flow cytometry analysis of expression of TRPC6 and CD34 in SVCs. Data are mean ± SEM. Statistical comparisons are done using Student's *t*-test for bar graphs. ***$p < 0.001$.

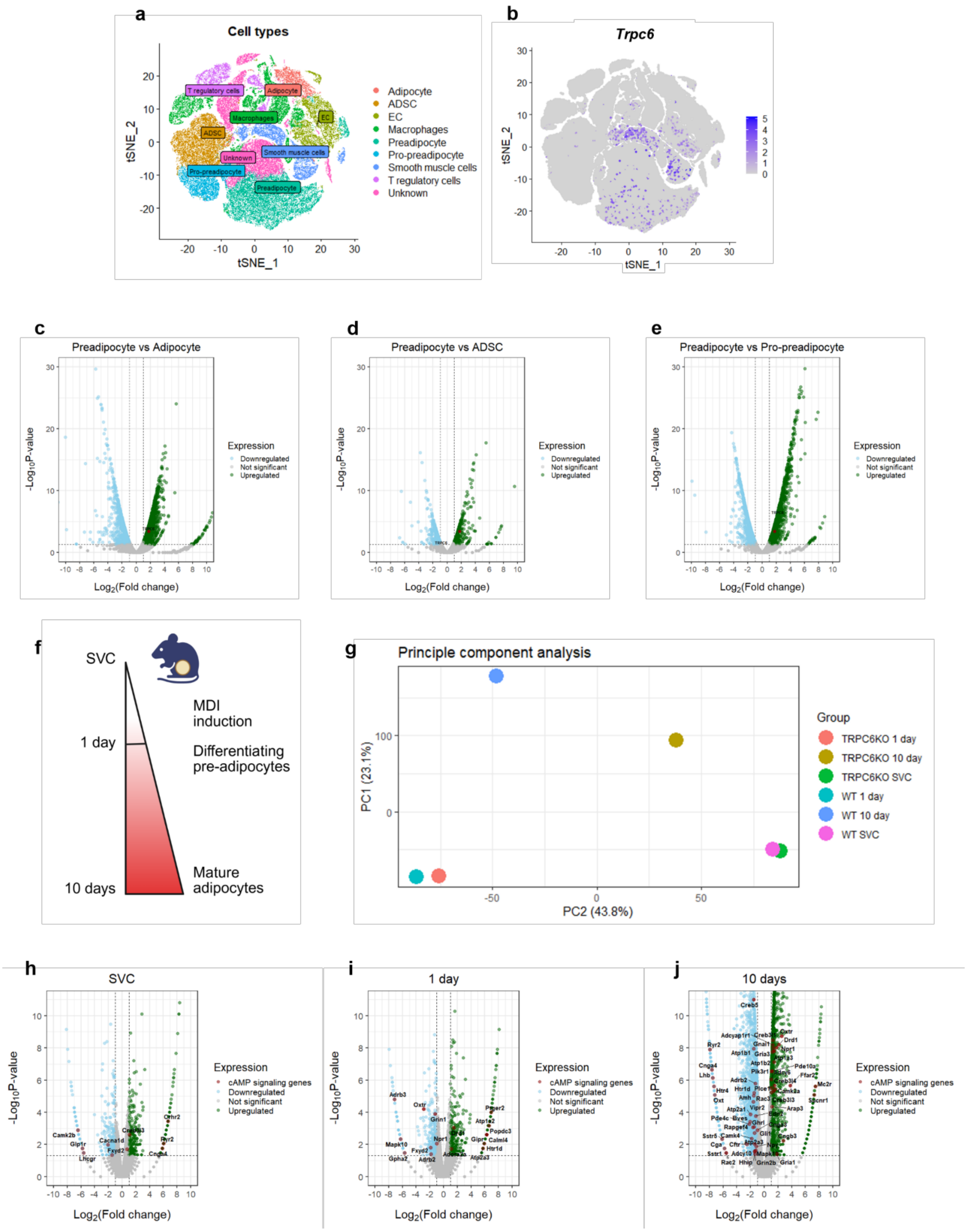
a
Cell types
tSNE_2
tSNE_1
T regulatory cells
Adipocyte
Macrophages
EC
ADSC
Smooth muscle cells
Unknown
Pro-preadipocyte
Preadipocyte
Adipocyte
ADSC
EC
Macrophages
Preadipocyte
Pro-preadipocyte
Smooth muscle cells
T regulatory cells
Unknown
b
Trpc6
tSNE_2
tSNE_1
c
Preadipocyte vs Adipocyte
d
Preadipocyte vs ADSC
e
Preadipocyte vs Pro-preadipocyte
-Log10P-value
Log2(Fold change)
Expression
Downregulated
Not significant
Upregulated
f
SVC
MDI induction
1 day
Differentiating pre-adipocytes
10 days
Mature adipocytes
g
Principle component analysis
PC1 (23.1%)
PC2 (43.8%)
Group
TRPC6KO 1 day
TRPC6KO 10 day
TRPC6KO SVC
WT 1 day
WT 10 day
WT SVC
h
SVC
i
1 day
j
10 days
-Log10P-value
Log2(Fold change)
Expression
cAMP signaling genes
Downregulated
Not significant
Upregulated

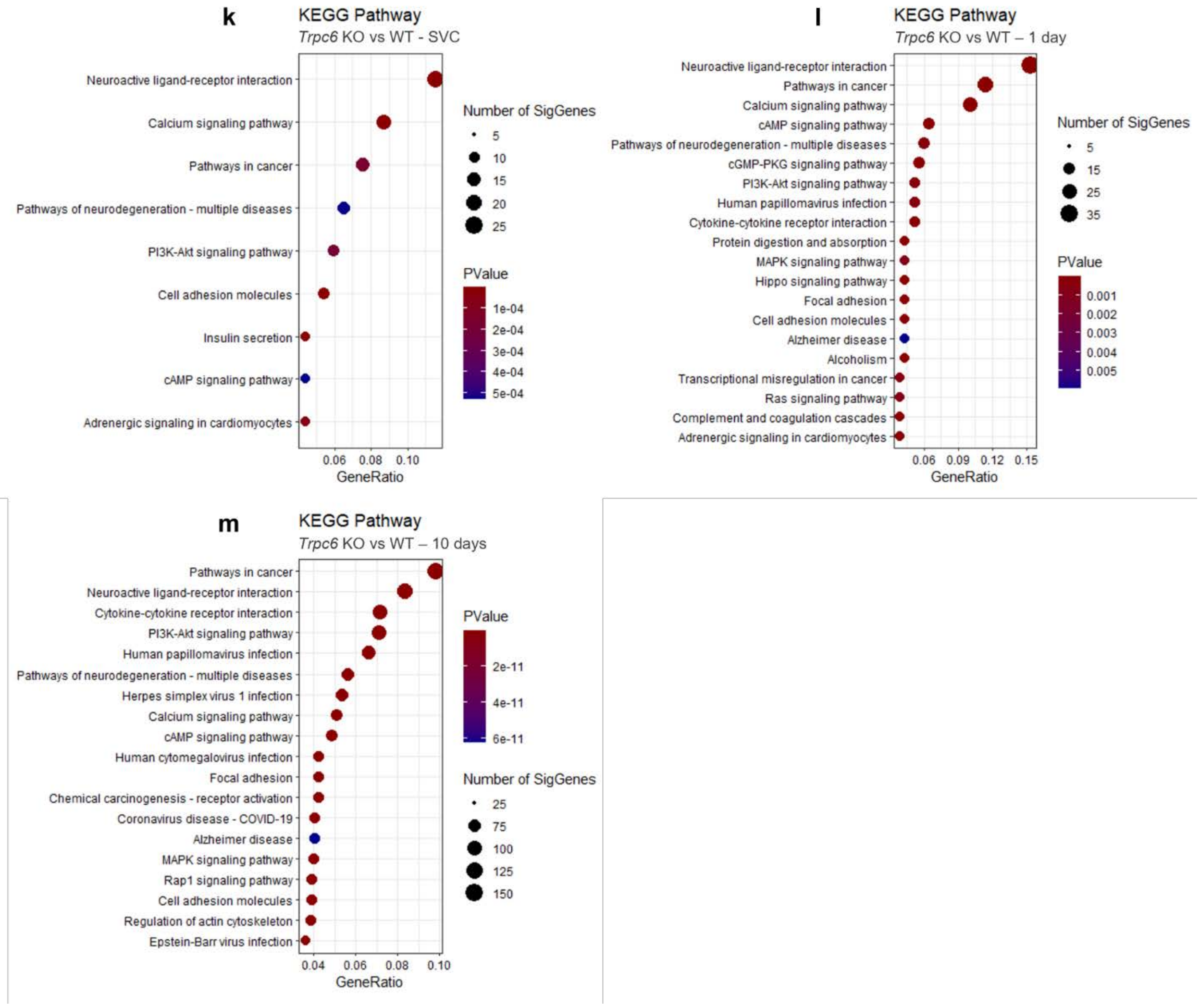

Supplementary Fig. 11 Transcriptomic profiling reveals Ca^{2+} and cAMP pathway dysregulation in Trpc6 KO progenitors. a *t*-distributed stochastic neighbor embedding plot of adipose-derived regenerative cells scRNA-seq with cell types (EC: endothelial cell, ADSC: adipose-derived stem cell) clusters annotated. **b** t-SNE plots showing the expression and distribution of *Trpc6*. **c-e** Volcano plot showing differentially expressed genes by pseudo-bulk RNA-seq analysis between Preadipocyte vs Adipocyte (**c**), Preadipocyte vs ADSC (**d**), and Preadipocyte vs Pro-preadipocyte (**e**). **f** Experimental design of analysis time-points for RNA sequencing of adipogenesis stages. **g** Principal component analysis of all samples. **h-j** Volcano plots of DEGs of *Trpc6* KO vs WT at different time points (SVC (**h**), 1 day (**i**), and 10 days (**j**). **k-m** KEGG pathway enrichment results of bulk RNA-Seq for *Trpc6* KO vs WT at different time points (SVC (**k**), 1 day (**l**), and 10 days (**m**). Statistical comparisons are performed using a two-way ANOVA for multiple-group analysis.

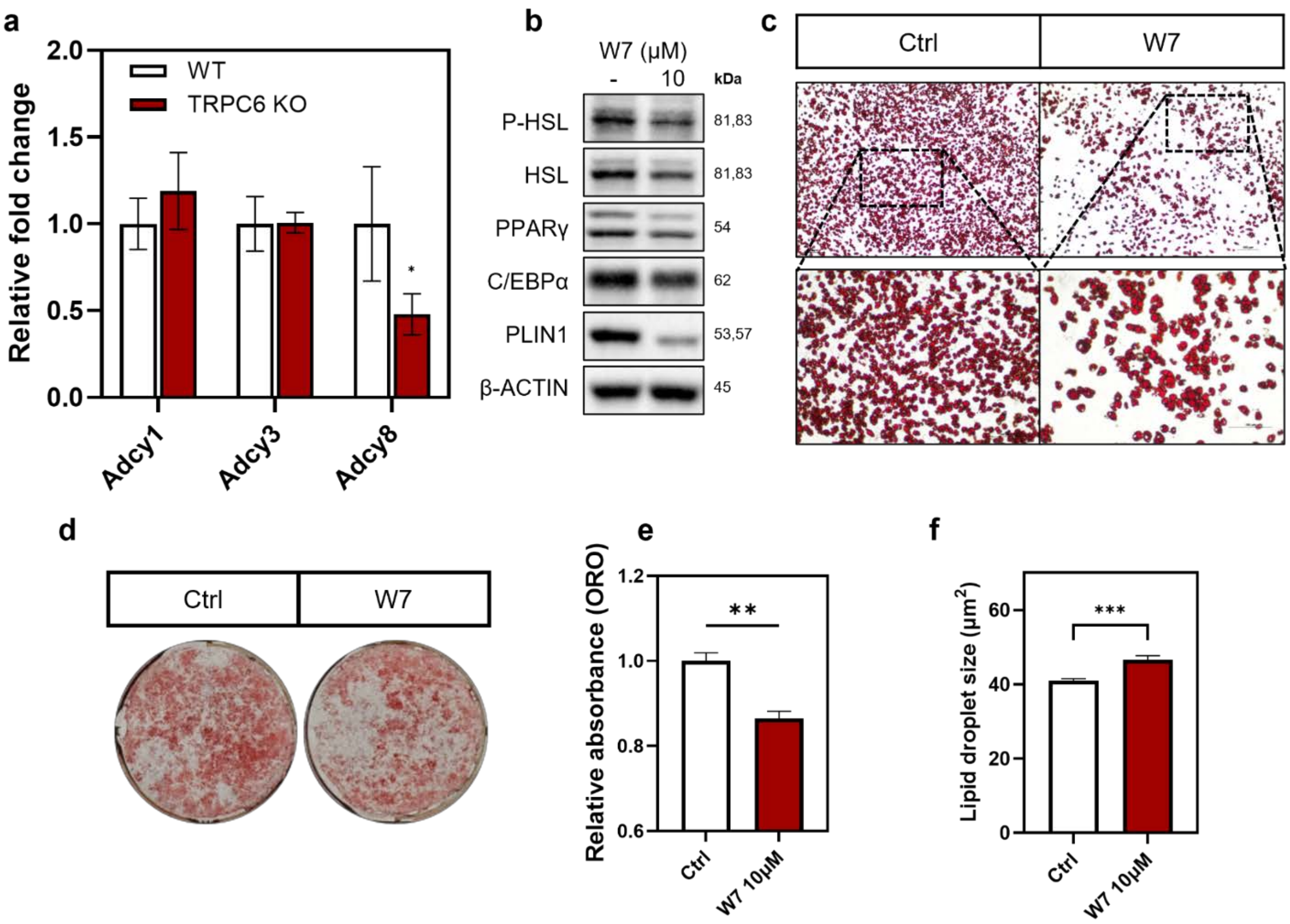

Supplementary Fig. 12 Adcy8 is altered in Trpc6 KO adipocyte, and its upstream inhibitor, W7, reduces adipogenesis and causes hypertrophic lipid droplet formation. a Relative mRNA expression of *Adcy1, Adcy3,* and *Adcy8* in WT and *Trpc6* KO differentiated primary white adipocytes (n = 9 per group). **b** Immunoblot and relative quantification of P-HSL, HSL, PPARγ, C/EBPα, and PLIN1 in Control (Ctrl) and W7-treated differentiated primary white adipocytes. **c-f** Representative Oil Red O (ORO) – stained images (**c**, **d**), quantified relative ORO absorbance (**e**), and lipid droplet size quantification (**f**) of Ctrl and W7 treated differentiated primary white adipocytes (n = 3 per group). Scale bar: 100 μm (n = 4 per group). Data are mean ± SEM. Statistical comparisons are done using Student's t-test for bar graphs. ns represent not significant, *$p < 0.05$, **$p < 0.01$, ***$p < 0.001$.

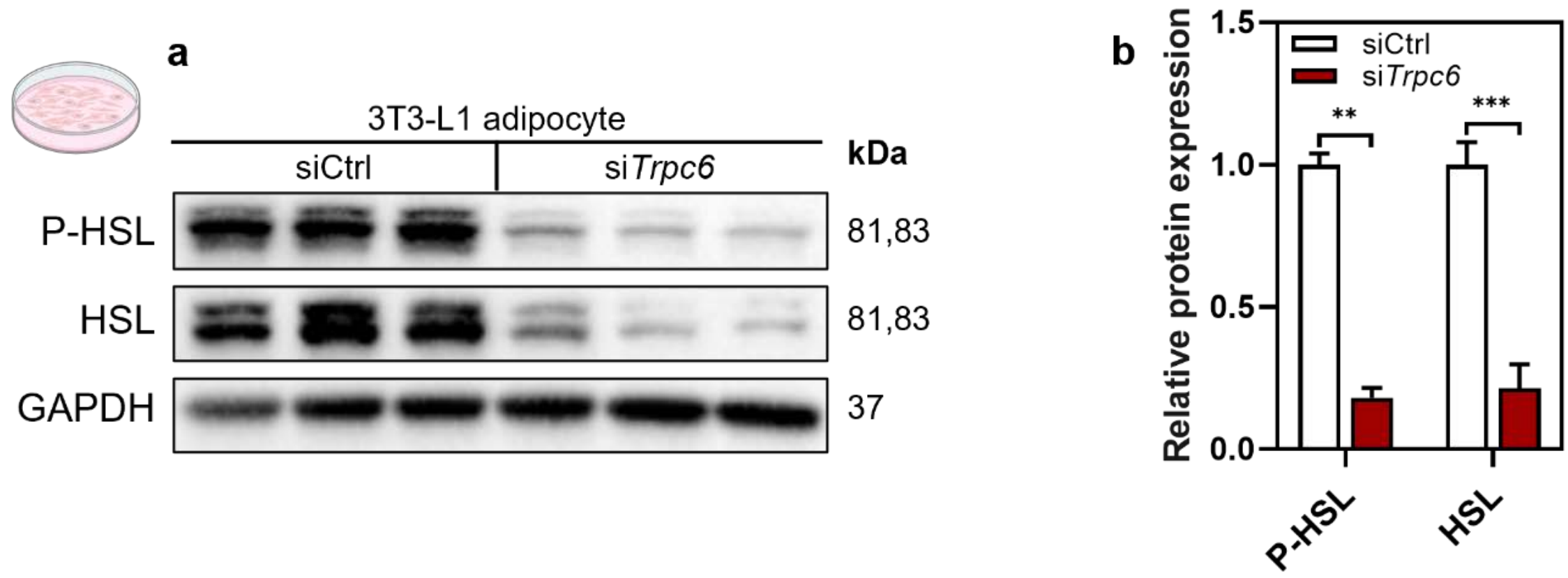

Supplementary Fig. 13 HSL activation is impaired in TRPC6-depleted 3T3-L1 adipocytes. **a**, **b** Immunoblots and relative quantification of P-HSL and HSL expression in control (siCtrl) and TRPC6 knockdown (siTRPC6) differentiated 3T3-L1 cells (n = 3 per group). Data are mean ± SEM. Statistical comparisons are done using Student's t-test for bar graphs. ** $p < 0.01$. *** $p < 0.001$.

Supplementary Table 1. List of antibodies.

Target antigen	Manufacturer	Catalog No.	Used dilution	Applications
**ACADVL**	Abcam	ab155138	1:1000	WB
**C/EBPα**	Cell Signaling	2295	1:1000	WB
**C/EBPβ**	Cell Signaling	3087	1:1000	WB
**CD34-Alexa Fluor® 488**	Santa Cruz	sc-74499 AF488	1:100	ICC and FACS
**CPT1A**	Cell Signaling	12252	1:1000	WB
**GAPDH**	Cell Signaling	5174	1:2000	WB
**DRP1**	Cell Signaling	5391	1:1000	WB
**MFF**	Cell Signaling	84580	1:1000	WB
**MFN1**	Abcam	ab126575	1:1000	WB
**HSL**	Cell Signaling	4107	1:1000	WB
**P-HSL**	Cell Signaling	4139	1:1000	WB
**Perilipin-1**	Cell Signaling	9349	1:1000	WB
**PPARγ**	Cell Signaling	2435	1:1000	WB
**OPA1**	Cell Signaling	80471	1:1000	WB
**STAT3**	Cell Signaling	9139	1:1000	WB
**p-STAT3**	Cell Signaling	9145	1:1000	WB
**Total OXPHOS Rodent Cocktail**	Abcam	ab110413	1:1000	WB
**TRPC6**	Alomone Lab	ACC017	1:500	WB
**TRPC6-Alexa Fluor® 594**	Santa Cruz	sc-515837 AF594	1:100	ICC and FACS
**β-actin**	Santa Cruz	sc-47778	1:2000	WB

Supplementary Table 2. Primer list.

Gene	Forward	Reverse
Trpc1	GATTTGCTCGCATACCTCGAAAG	TGTCGCATGGACGTCAGGTAG
Trpc3	ATCTGGAACTGGGCATGGGTAA	TGATATCGTGTTGGCTGATTGAGAA
Trpc4	AACCTGGTGAAGCGGTACGTG	ACTTCGAAGCGGAAGCTAGAAATG
Trpc5	GCAGCATTGTATGTGGCAGGA	CCTCGCCAAGGTTCATCTGAC
Trpc6	GACAAAAGTCACACTGGGGG	ACCACAGCGATTGCATAAAGA
Trpc7	TGGGCCTTCAGCACGTATCTC	AACCTGACAGCCAATAGCACCTTC
Ppary	TGTGAGACCAACAGCCTGAC	CACCGCTTCTTTCAAATCTTGTC
Adrb1	GCCCTTTCGCTACCAGAGTT	ACTTGGGGTCGTTGTAGCAG
Adrb2	CAATAGCAACGGCAGAACGG	TCAACGCTAAGGCTAGGCAC
Adrb3	TTGTCCTGGTGTGGATCGTG	TTGGAGGCAAAGGAACAGCA

Supplementary Table 3. Top 20 significantly enriched KEGG pathways in the SVC group Trpc6 KO vs WT.

**Map Name**	**No. of Common Genes**	***p*-value**	**FDR**
Neuroactive ligand-receptor interaction	22	1.15E-11	2.88E-09
Calcium signaling pathway	17	1.53E-10	1.92E-08
Insulin secretion	9	5.31E-07	4.44E-05
Cell adhesion molecules	11	2.68E-06	0.000168
Leishmaniasis	7	2.34E-05	0.001174
Adrenergic signaling in cardiomyocytes	9	4.45E-05	0.001863
Arachidonic acid metabolism	7	7.05E-05	0.002527
Inflammatory mediator regulation of TRP channels	8	8.54E-05	0.002679
Complement and coagulation cascades	7	0.000123	0.003388
Circadian entrainment	7	0.000155	0.003388
Glycerophospholipid metabolism	7	0.000155	0.003388
Pathways in cancer	15	0.000162	0.003388
PI3K-Akt signaling pathway	12	0.000191	0.003694
Oxytocin signaling pathway	8	0.000271	0.004748
African trypanosomiasis	5	0.000284	0.004748
Tight junction	8	0.00046	0.007212
Pathways of neurodegeneration - multiple diseases	13	0.000527	0.007413
cAMP signaling pathway	9	0.000532	0.007413
Carbohydrate digestion and absorption	5	0.000634	0.007954

Supplementary Table 4. Top 20 significantly enriched KEGG pathways in the 1-day differentiated group Trpc6 KO vs WT.

**Map Name**	**No. of Common Genes**	***p*-value**	**FDR**
Neuroactive ligand-receptor interaction	35	8.05E-22	2.24E-19
Calcium signaling pathway	24	5.22E-16	7.26E-14
Pathways in cancer	27	6.78E-11	6.29E-09
cAMP signaling pathway	16	1.05E-08	7.27E-07
cGMP-PKG signaling pathway	14	2.61E-08	1.45E-06
Protein digestion and absorption	11	1.55E-07	7.18E-06
Complement and coagulation cascades	10	4.91E-07	1.95E-05
Hippo signaling pathway	11	4.85E-06	0.000169
Renin secretion	8	1.21E-05	0.000368
Arrhythmogenic right ventricular cardiomyopathy	8	1.32E-05	0.000368
Cell adhesion molecules	11	1.78E-05	0.000449
ECM-receptor interaction	8	3.18E-05	0.000682
Adrenergic signaling in cardiomyocytes	10	3.19E-05	0.000682
Hypertrophic cardiomyopathy	8	3.95E-05	0.00077
Focal adhesion	11	4.15E-05	0.00077
Cytokine-cytokine receptor interaction	13	4.72E-05	0.000802
Alcoholism	11	4.91E-05	0.000802
Adipocytokine signaling pathway	7	8.41E-05	0.001299
Vascular smooth muscle contraction	9	0.000114	0.001672

Supplementary Table 5. Top 20 significantly enriched KEGG pathways in 10 days differentiated group Trpc6 KO vs WT.

**Map Name**	**No. of Common Genes**	***p*-value**	**FDR**
Neuroactive ligand-receptor interaction	113	1.71E-48	3.26E-46
Cytokine-cytokine receptor interaction	98	1.96E-48	3.26E-46
Pathways in cancer	131	1.38E-46	1.54E-44
PI3K-Akt signaling pathway	97	7.28E-39	6.06E-37
Human papillomavirus infection	91	8.42E-34	5.6E-32
Calcium signaling pathway	71	3.46E-31	1.92E-29
cAMP signaling pathway	68	1.24E-30	5.91E-29
ECM-receptor interaction	42	6.02E-28	2.5E-26
Protein digestion and absorption	45	7.15E-27	2.64E-25
Focal adhesion	59	8.71E-26	2.9E-24
Cell adhesion molecules	55	9.56E-25	2.89E-23
Complement and coagulation cascades	39	3.01E-23	8.34E-22
Chemical carcinogenesis - receptor activation	59	4.73E-23	1.21E-21
Rap1 signaling pathway	55	4.38E-21	1.04E-19
JAK-STAT signaling pathway	49	5.23E-21	1.16E-19
cGMP-PKG signaling pathway	49	8.99E-21	1.87E-19
Human cytomegalovirus infection	59	4.44E-20	8.7E-19
Coronavirus disease - COVID-19	57	2.32E-19	4.3E-18
Regulation of actin cytoskeleton	54	1.01E-18	1.77E-17

SUPPLEMENTARY MATERIALS AND METHODS

Animals

Trpc6 knockout mice have been previously described[1]. Adult male mice were used in this study. All mice were housed in individually ventilated cage (IVC) racks at a controlled temperature (22 ± 3°C) and humidity (50 ± 10%) under fluorescent lamp lights from 6:00 to 18:00. They were fed solid food 5L79 (LabDiet, St. Louis, MO, USA). All mice were maintained in pathogen-free barrier facilities, and their body weights were routinely checked and measured during the experiments. All animal experiments were conducted according to the eighth edition of the Guide for the Care and Use of Laboratory Animals (NRC 2011), and all experimental protocols involving mice were approved by the Institutional Animal Care and Use Committee (IACUC) at Yonsei University Wonju College of Medicine (YWC-191015-1).

Metabolic phenotyping

The mice were individually housed in a 12-chamber Comprehensive Laboratory Animal Monitoring System (CLAMS) (IT52SD, Columbus Instruments, OH, USA) to measure the metabolic rate. After a 6-hour acclimation, oxygen consumption (VO_2), carbon dioxide production (VCO_2), food intake (FI), and locomotor activity were assessed for each mouse. The respiratory exchange ratio (RER) was calculated as VCO_2/VO_2. Body fat and lean masses of mice were analyzed using an LF50 Body Composition Mice Analyzer (John Morris Group, Australia).

Glucose tolerance tests

The mice were fasted for 15 h before the experiment. Mice were intraperitoneally injected with 2 mg of glucose per gram of body weight. Blood glucose levels were measured using tail blood samples and a blood glucose testing kit (AGM-4100, All Medicus) at 0, 15, 30, 45, 60, 90, and 120 min after injection.

Insulin tolerance tests

The mice were fasted for 6 h before the experiment because of the risk of severe hypoglycemia and death. The rats were injected with 0.75 IU of insulin per gram of body weight. Blood glucose levels were measured using tail blood samples and a blood glucose testing kit at 0, 15, 30, 45, 60, 90, and 120 min after the injection.

In vivo temperature measurement

Mice were subjected to measure fasting temperature for 6 h. Skin temperatures were measured after fasting was completed, using a thermal imaging camera (FLIR T530). Taken images were analyzed using ThermImageJ plugin of Fiji software. Rectal temperatures were measured every hour during fasting time using high TESTO DT-08S-M high-precision rectal thermometer.

Cell culture

Immortalized mouse 3T3-L1 preadipocytes (ATCC) were cultured in Dulbecco's modified Eagle's medium (DMEM) with high glucose (Hyclone, Cytiva) supplemented with 10% (v/v) newborn calf serum (CS, Gibco®) and 1% penicillin/streptomycin (P/S, Gibco®). Adipogenesis was induced using an MDI cocktail containing 2 μM dexamethasone, 0.5 mM 3-isobutyl-1-methylxanthine (IBMX), and 10 μg/mL Insulin in DMEM high glucose with 10% (v/v) fetal bovine serum (FBS, Gibco®) and 1% P/S to culture the cells for 2 days. Subsequently, the culture medium was changed to insulin-containing medium every 2 days until 6 days of adipogenesis was achieved.

Treatment of Rotenone (5 nM), W7 (10 μM) or SS-31 (1 μM) were applied at the same time with the start of adipogenesis induction to assess their effect on adipocyte differentiation.

Primary cell isolation

The primary white and brown adipocyte isolation protocol was adapted from a previous publication[2]. Inguinal WAT and scapular BAT were removed from 5- to 8-week-old mice. Processed tissues were minced into small pieces in lysis solution (with Collagenase II and DNase I (10104159001, Roche®)) and incubated in a shaking incubator at 37 °C for 30 min. The lysate was then filtered through a 100 μm cell strainer (542000, Greiner). The filtered solution was washed and centrifuged to collect the cell pellet, followed by treatment with ACK lysis buffer (A1049201, Gibco®) to remove red blood cells. Finally, the isolated cell pellets were plated on a culture plate and then changed to fresh medium after 2 h to remove debris. Primary adipocytes

were cultured in DMEM with high glucose supplemented with 15% (v/v) FBS and 1% P/S.

Cells were induced to differentiate into primary white adipocytes using 15% DMEM with high glucose, containing the same MDI mixture as the 3T3-L1 cell line. The differentiation medium was changed after 2 days and maintained for 4 days. Subsequently, the induced cells were cultured in DMEM supplemented with 15% FBS, 1% P/S, and 10 μg/mL insulin, with a medium refresh every 2 days until day 6. Primary brown adipocytes were differentiated in DMEM supplemented with 15% FBS, 1% P/S, 10 μg/mL insulin (CM), 10 μM rosiglitazone, and 125 μM indomethacin, with a medium refresh every 2 days until day 6.

Flow cytometry analysis

Isolated primary cells were stained with fluorescent conjugated primary antibody at the vendor-recommended dilution. Then they were washed and diluted in PBS to reach a concentration of 1 x 10^6 cells/ml and measured using a fluorescence-activated cell sorting system (FACSAria III, BD Biosciences, USA). Alexa 594 fluorescent were measured using PE-TexasRed channel and Alexa 488 fluorescent was measured using FITC channel.

Co-immunostaining

Cells were seeded on Poly L-lysine coated coverslips overnight and then subjected to process of fixing with 4% PFA and washing with PBS. Then the coverslips are blocked

using UltraCruz® Blocking Reagent (sc-516214, Santa Cruz) for 1 h. Prepared samples were then co-stained with fluorescent conjugated primary antibodies targeting TRPC6 and CD34 overnight. Finally, the coverslips were mounted on microscope slide using VECTASHIELD® Antifade Mounting Medium (H-1000, Vector Laboratories) and sealed using nail polish.

Metabolic flux assays

The oxygen consumption rate (OCR) and extracellular acidification rate (ECAR) were measured using an Extracellular Flux Analyzer XF-96 (Agilent, Santa Clara, CA, USA). Terminally differentiated adipocytes were seeded at a confluence of 12,000 cells/well in a Seahorse 96-well plate containing CM and incubated overnight. On experiment day, the medium is replaced with XF-DMEM pH 7.4 (103575-100, Agilent, USA) containing 1 mM pyruvate, 2 mM L-glutamine, and 10 mM glucose for OCR measurements, or with 2 mM L-glutamine only for ECAR measurements, at least 1 h before the measurement.

OCR was measured using the Seahorse XF Cell Mito Stress Test Kit (103015-100, Agilent, USA) with 1.5 μM oligomycin, 1.5 μM FCCP, and 1.25 μM rotenone/antimycin A. ECAR was assayed by the XF Glycolysis Stress Test Kit (103020-100, Agilent, USA) with 1.5 mM glucose, 1.5 μM oligomycin, and 50 mM 2-DG. The measurements were performed according to the manufacturer's instructions.

Electrophysiology measurement

TRPC6 currents were recorded in HEK293FT cells overexpressing TRPC6 using the patch-clamp technique, as described previously[3]. Whole-cell currents were measured under a whole-cell dialyzed configuration using an EPC-10 patch-clamp amplifier (Heka Electronik, Lambrecht, Germany). The patch electrodes were coated with a silicone elastomer (Sylgard 184; Dow Corning, Midland, MI), fire-polished, and had resistances of 2-3 MΩ when filled with the pipette solution. The cell membrane capacitance and series resistance were electronically compensated (> 80 %) using an EPC-10 amplifier. Data were acquired using Pulse/Pulsefit (v8.50) software (Heka Electronik). All electrophysiological recordings were performed at room temperature (approximately 20-24°C). The pipette and bath solution contained (in mM) 120 Cs-aspartate (Cs-Asp), 10 CsCl, 1 $MgCl_2$, 2 MgATP, 5 EGTA, 1.5 $CaCl_2$ (free [Ca^{2+}] =70 nM), and 10 HEPES (pH 7.2), and 140 Na-aspartate, 5 KCl, 0.5 EGTA, and 10 HEPES (pH 7.4), respectively.

Intracellular cAMP level assay

Cells were harvested, lysed in 0.1 M HCl and centrifuged to collect supernatant for analysis. cAMP levels were measured using a direct cAMP ELISA kit (Enzo Life Sciences, Farmingdale, NY, USA), following the manufacturer's instructions. The absorbance was measured at 405 nm using a microplate reader.

Serum leptin measurements

Retro-orbital sinus blood samples were immediately collected in collection tubes (365967, BD Biosciences) and allowed to clot for at least 30 min at room temperature before centrifugation at 2,000 × g for 15 min at 4 °C. The collected serum was transferred to a new tube and stored at -80 °C prior to measurement. Serum leptin levels were measured using an ELISA kit (KMC2281, Thermo Fisher Scientific, Waltham, MA, USA) according to the manufacturer's protocol.

Immunohistochemistry.

Tissues were collected from 8-week-old and 20-week-old mice. Fat depots, liver, and skeletal muscle tissues were fixed in 4% paraformaldehyde in PBS (ST4090PP, Supreme Tech, Korea) for at least 48 hours at room temperature. Following fixation, the tissues were dehydrated in graded ethanol concentrations (70%, 80%, 90%, 95%, and 100%), cleared in xylene, and embedded in paraffin. Sections of 4 µm thickness were mounted on glass slides at 45 °C, dewaxed with xylene, rehydrated from graded ethanol (70 %, 80 %, 90 %, 95 %, and 100% to distilled water), and stained with hematoxylin and eosin at room temperature. The adipocyte cell size and lipid droplet size were quantified using Fiji software.

Immunoblot

Cells and tissues were lysed using RIPA lysis and extraction buffer (Thermo Scientific), containing cOmplete EDTA-free protease inhibitor cocktail (Roche) and PhosSTOP

(Roche). The lysates were centrifuged at 17,000 × g for 20 min at 4 °C. The protein concentration of each sample was measured using the Pierce™ BCA Protein Assay Kit (Thermo Fisher Scientific), following the manufacturer's protocol. Samples were denatured in Elpis Biotech Laemmli's 5x sample buffer and loaded onto an SDS-PAGE gel with adjusted volume to achieve an equal amount of protein per well. The blots were visualized using a ChemiDoc XRS+ system (Bio-Rad Laboratories, Hercules, CA, USA).

Live cell imaging

All live-cell imaging-based methods utilized a flow-based system with temperature and humidity control as described previously[4]. A normal physiological saline solution (NPSS) that contained (in mM) 135 NaCl, 5 KCl, 1 $MgCl_2$, 2 $CaCl_2$, 10 HEPES, and 5.5 glucose (pH 7.4) was used as the base-flowing buffer for all measurements. Before live imaging, all cells were seeded on Poly-L-Lysine-coated 12 mm coverslips (111520; Paul Marienfeld, Germany). The intracellular Ca^{2+} rise was measured using the Fura-2AM Ca^{2+} indicator. Briefly, cells were loaded with 5 μM Fura-2AM (F1221, Thermo Scientific) in NPSS for 1 h in a 5% CO2 incubator at 37 °C before measurement. Fura-2 signals were obtained by alternating excitation at 340 or 380 nm and emission at 510 nm.

TRPC6-dependent systolic Ca^{2+} rise was measured using Fura-2AM. Briefly, cells were loaded with 5 μM Fura-2AM dye for 1 h before measurement. TRPC6 activators, Endothelin-1 (100 nM) or OAG (100 μM), were directly applied during live-cell imaging to measure follow up Ca^{2+} rise.

Intracellular cAMP levels were measured by delivery of Epac-S-H187 (a cAMP biosensor, Addgene, RRID: Addgene_170348) vector to the cell by transfection 48 h before measurement. A single excitation at 435 ± 20 nm and double emissions at 482 ± 18 nm and 530 ± 20 nm were applied to record the FRET signals. For TRPC6-dependent cAMP response measurements, Flag-TRPC6 and hM3R were co-transfected with the Epac-S-H187 vector. All live cell imaging data were obtained using MetaFluor software (Sutter Instruments) and analyzed using Fiji software.

Long-chain fatty acid respiration measurement

Long-chain fatty acid respiration was done as described[5]. Briefly, cells were seeded at a confluence of 12,000 cells/well in a Seahorse 96-well plate 96-well 48 hours before the measurement. One day before the measurement, cells were maintained in a substrate-limited medium (XF-DMEM, pH 7.4 (103575-100, Agilent, USA), 0.5 mM glucose, 1 mM GlutaMAX, 0.5 mM Carnitine, 1% FBS). On the examination day, cells are washed and subjected to FAO assay buffer (in mM: 1 NaCl, 4.5 KCl, 1.25 $CaCl_2$, 2 $MgSO_4$, 1.2 NaH_2PO_4, 2.5 glucose, 0.5 carnitine, and 5 HEPES at pH = 7.4). Etomoxir (0.4 mM) and/or vehicle treatment (DMSO) were applied 15 minutes before the test, and right before the test, 1 mM palmitate and/or 0.17 mM BSA were added to the cell to activate FAO. Mitochondrial oxygen consumption was measured as previously described.

Mitochondria membrane potential measurement

The cells were seeded in 96-well microplates (655090; Greiner, Austria). Before measurement, cells were washed with Krebs-Ringer bicarbonate buffer (KRB) and loaded with 500 nM lipophilic cationic dye 5,5',6,6' tetrachloro-1,19,3,39-tetraethylbenzimidazolyl-carbocyanine iodide (JC-1) (T3168, Molecular Probes, Thermo Fisher Scientific) in KRB for 30 minutes. The fluorescent intensity was measured using a fluorescent microplate reader (Flexstation II, Molecular Devices) with dual excitation / double emission (540/590 nm and 490/535 nm, respectively, for mitochondrial aggregated JC-1 and cytosolic JC-1 signals). Total depolarization of the mitochondrial membrane potential was achieved by application of 10 μM carbonyl cyanide 4-(trifluoromethoxy) phenylhydrazone (FCCP).

Mitochondrial ROS measurement

Cells were seeded on Poly-L-Lysine-coated 12 mm coverslips and loaded with 500 nM MitoSOX (M36008, Invitrogen, USA) in KRB for 15 min in a CO_2 incubator. Images were taken at an excitation wavelength of 510 nm and an emission wavelength of 580 nm using MetaMorph software and analyzed using Fiji software.

Lipid staining

Mature adipocytes (six days post-induction) were fixed with 4% Paraformaldehyde for 15 min at room temperature. The fixed cell layer was semi-dried by washing with 60%

isopropyl alcohol and then stained with an Oil Red O solution (0.125 ml/cm²) or BODIPY (D3922, Invitrogen™) solution (1 μM) in PBS.

For Oil Red O staining, the stained cell plate was washed three times with clean water, and pictures were taken using a bright-field microscope. For quantification, 100% isopropyl alcohol was used to elute the stained dye (0.25 ml/cm²), and the optical density was measured at 510 nm. Surviving cells were normalized using Crystal Violet staining (following the same protocol as ORO staining) and quantified by elution with a 100% methanol solution.

For BODIPY staining, the stained cell coverslips were washed three times with cold PBS and counterstained with DAPI (1 μg/ml) in PBS. The samples were then washed three times with cold PBS. Finally, the coverslips were mounted to microscope slides using VECTASHIELD® Antifade Mounting Medium (H-1000, Vector Laboratories) and sealed with nail polish. Images were acquired using the LSM-800 confocal microscope and analyzed using Fiji.

Transmission electron microscopy

Tissue slices were fixed in 0.1 M cacodylate buffer at 4 °C, washed in 0.1 M cacodylate buffer, and then post fixed in 1% osmium tetroxide in the same buffer for 90 min at 4 °C. Specimens were dehydrated through a graded ethanol series, exchanged with propylene oxide, and embedded in an Epon mixture. Ultrathin sections (70 nm) were obtained using an ultramicrotome (Reichert Jung, Vienna, Austria) with a diamond knife. Ultrathin sections were double-stained with 2.5% uranyl acetate for 15 min and 2% lead citrate for 2 min 30 s, and an HT7800 transmission electron microscope

(Hitachi, Tokyo, Japan) was used for image acquisition. Transmission electron microscopy images were analyzed using the Fiji software, as described before[6].

RT-PCR and quantitative PCR.

Tissue or cell samples were lysed using RiboEx (301-902, Geneall Biotechnology, Korea). Reverse transcription was performed to obtain complementary DNA using ReverTra Ace™ qPCR RT Master Mix with a gDNA remover (FSQ-301, Toyobo, Japan) following the manufacturer's protocol. Quantitative PCR was performed using *Power* SYBR Green PCR Master Mix (4367659, Applied Biosystems) according to the manufacturer's protocol on a real-time PCR system. Comparative Ct was used for the analysis. The primer sequences used for quantitative real-time PCR are listed in Supplementary Table 2.

RNA-sequencing and analysis

iWAT-derived SVF cells from WT and *Trpc6* KO mice were cultured and differentiated in a 12-well plate. Three time points were defined for bulk-RNAseq analysis: native SVF cells, MDI-induced cells after 1 day, and fully differentiated cells after 10 days of maintenance following initial induction.

RNA from SVF cells was extracted using RiboEx (301-902, Geneall Biotechnology, South Korea) and sent to Macrogen (Seoul, South Korea) for transcriptome sequencing on the Illumina NovaSeq platform. Before analysis, Trimmomatic v0.38 was employed to eliminate adapter sequences and trim low-quality

bases, and the data were then aligned to the Mus musculus genome (mm10) using HISAT v2.1.0[7]. The aligned data (SAM file format) were sorted and indexed using SAMtools v1.9. In the subsequent alignment, the transcripts were assembled and quantified using StringTie v2.1.3b[8].

Differential gene expression analysis between the two conditions was conducted using edgeR v3.40.2[9]. GProfiler[10] was used for enrichment analysis of candidate gene sets. KEGG database[11] was used as the reference pathway. All data analyses and visualization of differentially expressed genes were performed using R 4.2.2 (www.r-project.org).

REFERENCES

1. Dietrich, A. et al. Increased vascular smooth muscle contractility in TRPC6-/- mice. *Mol. Cell. Biol.* **25,** 6980–6989 (2005).
2. Hearnden, R., Sandhar, B., Vyas, V. & Longhi, M. P. Isolation of stromal vascular fraction cell suspensions from mouse and human adipose tissues for downstream applications. *STAR Protoc.* **2,** 100422 (2021).
3. Xie, J. et al. Cardioprotection by Klotho through downregulation of TRPC6 channels in the mouse heart. *Nat. Commun.* **3,** 1238 (2012).
4. Kim, J. H. et al. Klotho may ameliorate proteinuria by targeting TRPC6 channels in podocytes. *J. Am. Soc. Nephrol.* **28,** 140–151 (2017).
5. Angelini, A., Pi, X. & Xie, L. Evaluation of long-chain fatty acid respiration in neonatal mouse cardiomyocytes using SeaHorse instrument. *STAR Protoc.* **3,** 101392 (2022).
6. Lam, J. et al. A universal approach to analyzing transmission electron microscopy with ImageJ. *Cells* **10,** 2177 (2021).
7. Kim, D., Langmead, B. & Salzberg, S. L. HISAT: a fast spliced aligner with low memory requirements. *Nat. Methods* **12,** 357–360 (2015).
8. Pertea, M. et al. StringTie enables improved reconstruction of a transcriptome from RNA-seq reads. *Nat. Biotechnol.* **33,** 290–295 (2015).
9. Robinson, M. D., McCarthy, D. J. & Smyth, G. K. edgeR: a Bioconductor package for differential expression analysis of digital gene expression data. *Bioinformatics* **26,** 139–140 (2010).
10. Raudvere, U. et al. g:Profiler: a web server for functional enrichment analysis and conversions of gene lists (2019 update). *Nucleic Acids Res.* **47,** W191–W198 (2019).

11. Kanehisa, M. & Goto, S. KEGG: kyoto encyclopedia of genes and genomes. *Nucleic Acids Res.* **28,** 27–30 (2000).
